# SNHG16 upregulation-induced positive feedback loop with YAP1/TEAD1 complex in Colorectal Cancer cell lines facilitates liver metastasis of colorectal cancer by modulating CTCs epithelial-mesenchymal transition

**DOI:** 10.7150/ijbs.73438

**Published:** 2022-08-16

**Authors:** Zhenxian Xiang, Guoquan Huang, Haitao Wu, Qiuming He, Chaogang Yang, Rongzhang Dou, Qing Liu, Jialing Song, Yan Fang, Shuyi Wang, Bin Xiong

**Affiliations:** 1 Department of Gastrointestinal Surgery, Zhongnan Hospital of Wuhan University, No.169 Donghu Road, Wuchang District, Wuhan, 430071, China.; 2 Department of Gastric and Colorectal Surgical Oncology, Zhongnan Hospital of Wuhan University, No.169 Donghu Road, Wuchang District, Wuhan, 430071, China.; 3 Hubei Key Laboratory of Tumor Biological Behaviors, No.169 Donghu Road, Wuchang District, Wuhan, 430071, China.; 4 Hubei Cancer Clinical Study Center, No.169 Donghu Road, Wuchang District, Wuhan, 430071, China.; 5 Department of Thyroid and Breast Surgery, Maternal and Child Hospital of Hubei Province, Wuhan, 430071, People's Republic of China.; 6 Department of Gastrointestinal Surgery, Central Hospital of Enshi Tujia and Miao Autonomous Prefecture, Enshi, China.

**Keywords:** SNHG16, YAP1, TEAD1, CTCs, EMT

## Abstract

Circulating tumor cells (CTCs) are important precursors of colorectal cancer (CRC) metastasis. The epithelial-mesenchymal transition (EMT) process facilitates CTC invasion by allowing these cells to evade antimetastatic checkpoints to mediate distant metastasis. However, the specific molecular mechanism of tumor EMT remains largely unknown. Based on our previous research on the YAP1 pathway, we further studied the upstream molecule small nucleolar RNA host gene 16 (SNHG16), whose expression was correlated with advanced TNM stage, distant metastasis, and poor prognosis in CRC patients. Furthermore, loss- and gain-of-function assays revealed that SNHG16 promoted CRC colony formation, proliferation, migration, invasion, EMT, mesenchymal-like CTC generation, and liver metastasis through YAP1. Mechanistically, SNHG16 acted as a miRNA sponge to sequester miR-195-5p on Ago2, thereby protecting YAP1 from repression. Moreover, YAP1 bound TEA domain transcription factor 1 (TEAD1) to form a YAP1/TEAD1 complex, which in turn bound two sites in the promoter of SNHG16 and regulate SNHG16 transcription. Finally, *in vivo* experiments showed that the inhibition of SNHG16 suppressed tumor progression, and that YAP1 rescued the effect of SNHG16 on tumor progression. Herein, we have clarified a hitherto unexplored SNHG16-YAP1/TEAD1 positive feedback loop, that may be a candidate target for CRC treatment.

## Introduction

Colorectal cancer (CRC) is the third most common malignancy and the second leading cause of cancer-related mortality worldwide 1. To date, metastasis remains the leading cause of global cancer-related mortality 2. The liver is the most common site of CRC distant metastasis 3-5, and liver metastasis is the main cause of CRC-related death 6. Metastasis is a complicated process involving multiple steps that is driven by multiple mechanisms 7-9, and accumulative evidence has demonstrated that circulating tumor cells (CTCs) are the precursors of metastases 10. The epithelial-to-mesenchymal transition (EMT) process, which supports the release of CTCs and provides CTCs with several prometastatic traits 11, 12, is also involved in the whole metastasis process 13. Generally, EMT facilitates the release of CTCs at the beginning of metastasis 14. Furthermore, epithelial-like CTCs (^E^CTCs) can gain more mesenchymal traits to increase their invasive ability via EMT 15, thereby overcoming antimetastatic bottlenecks 16, 17 and achieving great potential for metastasis 18-23. In the EMT process, the expression of E-cadherin is inhibited, while the expression of N-cadherin and Vimentin is enhanced 24-26. Accumulative evidence has also revealed that the number of CTCs, especially mesenchymal-like CTCs (^M^CTCs) 27, is correlated with tumor metastasis 28, 29. However, further illumination of the molecular mechanism of EMT is needed to better understand the mechanisms of CRC liver metastasis, which will shed light on change in the CTCs type and further reveal the in-depth mechanism of CRC liver metastasis.

The activation of EMT stems from the interplay of many different factors, including the Hippo signaling pathway 30-33. As a master regulator of EMT 22, the abnormal expression of yes-associated protein 1 (YAP1) promotes malignant tumor proliferation and metastasis, induces EMT and cause drug resistance 22, 34-36. On the other hand, in its active form, YAP1 can function as a transcriptional coactivator predominantly mediated by its interaction with TEAD 37. MiR-195-5p has been demonstrated to be a tumor suppressor in various cancers 38. In our previous study, we demonstrated that miR-195-5p could potently inhibit the EMT process via YAP1 in CRC 39. However, we did not find a significant change in miR-195-5p expression when we upregulated or downregulated YAP1 expression. Therefore, further exploration of the upstream components of the miR-195-5p/YAP1 axis is important for further clarifying the mechanism of CRC EMT.

Long noncoding RNAs (lncRNAs), transcripts longer than 200 nucleotides with no or limited protein-coding potential 40, have also been implicated in EMT activation 41. The complicated secondary structure of lncRNAs allows lncRNAs to drive many important cellular phenotype changes in tumors through the interactions of lncRNAs with RNA and other components 40, 42. Consequently, lncRNAs can regulate EMT in a variety of ways 43, 44. For instance, lncRNAs can function as microRNA (miRNA) sponges to sequester miRNAs from endogenous target mRNAs, thus affecting tumor progression 45. Among the many cancer-related lncRNAs, small nucleolar RNA host gene 16 (SNHG16) is located mainly in the cytoplasm and was initially identified as an oncogene in neuroblastoma 46. LncRNA SNHG16 was demonstrated to regulate cancer cellular proliferation, invasion, EMT, and chemoresistance 47, 48; moreover, SNHG16 regulated the migration, invasion, and lipid metabolism of CRC cells, and therefore played pivotal roles in CRC progression 49. Bioinformatics analysis also found that lncRNA SNHG16 was one of the most upregulated lncRNAs in CRC. Intriguingly, using bioinformatics prediction, we also found the same miR-195-5p response elements in SNHG16 and YAP1. Therefore, whether lncRNA SNHG16 can act upstream of miR-195-5p, and thus regulate YAP1 expression by posttranscriptional modification, deserves to be further explored. More importantly, it remains largely unknown whether YAP1, a critical transcriptional coactivator, can activate the transcription of SNHG16.

The present study found that SNHG16 was upregulated in CRC and significantly associated with a poor prognosis in CRC patients. Cox-regression analysis revealed that SNHG16 was an independent prognostic biomarker in CRC. The results of loss- and gain-of-function analyses showed that SNHG16 can regulate CRC proliferation, migration, invasion, EMT, ^M^CTC generation, and liver metastasis. We also demonstrated that YAP1 was a functional mediator of SNHG16 and the oncogenic effect of SNHG16 was dependent on YAP1. Mechanistically, SNHG16 acted as a miRNA sponge to sequester miR-195-5p on Ago2, thereby protecting YAP1 from repression. Moreover, YAP1 bound TEAD1 to form a complex, which in turn bound the promoter of SNHG16 to regulate its transcription. Herein, we have clarified a previously unexplored positive feedback loop involving SNHG16 and YAP1/TEAD1. Through this positive feedback loop, SNHG16 promotes CTC EMT and CRC liver metastasis. These findings provide a novel mechanism of CRC liver metastasis and indicate that this feedback loop may be a candidate target in CRC treatment.

## Methods and materials

### CTC isolation and identification

The isolation and enrichment of CTCs were performed by a CTCBIOPSY device (Wuhan YZY Medical Science and Technology Co., Ltd., Wuhan, China), which was described in our previous research 50. According to the manufacturer's instructions, we diluted 1 ml mouse blood into 5 ml of 0.9% sodium chloride solution, and the total liquid was then transferred to ISET tubes with an eight μm diameter aperture membrane. Through positive pressure from 12 to 20 mmHg in ISET tubes, candidate CTCs were adhered to the ISET tube membrane and identified by three-color immunofluorescence.

### RIP assay

According to the instructions of the Magna RIP RNA-Binding Protein Immunoprecipitation Kit (Millipore, Billerica, MA, USA), we performed RIP to investigate the binding of miRNA-195-5p to lncRNA SNHG16. Anti-Ago2 antibodies (CST, USA) were used for immunoprecipitation, and the same species anti-IgG antibodies and total RNA (input controls) were used as controls. The co-precipitated RNAs were reverse transcribed to cDNA and detected by qRT-PCR.

### Co-IP

CRC cells were lysed in RIPA buffer and the protein concentration of lysates was quantified using the BCA reaction. After impurities were removed (referred to protein extraction), the clarified lysates were incubated with rabbit anti-YAP1 or anti-TEAD1 overnight with end-over-end shaking at 4°C. Subsequently, we used the Protein A/G Sepharose beads to capture the antigen-antibody complexes at 4°C for 12 h. Then, the beads were washed with PBS, and the complexes were boiled to separate antigens and antibodies from the beads. After elution from the beads, the expression levels of YAP1 and TEAD1 in the IP were analyzed by western blotting (WB).

### Pulldown assay with biotinylated miRNA

To assay whether miR-195-5p binds to lncRNA SNHG16, a biotin-avidin pulldown system was applied. Biotinylated miR-195-5p was synthesized by RioBio (RiboBio Co. Ltd., China), we called it miR-195-5p-Bio. Then, biotinylated miR-195-5p with mutated SNHG16 binding sites was also synthesized in RiboBio, termed miR-195-5p-mut-Bio. We also used NC-Bio as a negative control (NC) to ensure the accuracy of the result, and the operation process was as follows. Briefly, CRC cells were transfected with biotinylated miR-195-5p with the aid of Lipofectamine 2000 and then were rinsed and lysed in buffer 48 h later. Then, the cell lysates were treated with streptavidin magnetic beads, and the bound RNAs were isolated by TRIzol LS reagent for further RT-PCR analysis.

### ChIP

We performed ChIP using the Simple ChIP ® Enzymatic Chromatin IP Kit (ChIP) Kit (Cell Signaling, #9003, USA) according to the manufacturer's instructions. The first step is to lyse the tumor cells and crosslink proteins to DNA. Subsequently, the lysates of first step were sonicated into 300 bp to 600 bp fragments and immunoprecipitated with anti-TEAD1 antibody (Abcam, UK). In addition, we used normal rabbit immunoglobulin G (IgG) as a negative control. Then, we performed reverse crosslinking and DNA purification. Finally, the precipitated DNA was analyzed by qRT-PCR via SYBR-Green PCR Master Mix (Vazyme, Nanjing, China). Primers used for SNHG16 promoter regions are shown in the Supplementary Table 1.

### GST pulldown assay

To test whether YAP1 could interact with TEAD1 directly, we performed the GST pull-down assay as follows. First, after the transformation, cloning and expression of the GST-YAP1 fusion protein in E. coli, the GST-tagged YAP1 fusion protein was further purified using glutathione-Sepharose 4B beads. For the pull-down assay, the GST or GST-tagged YAP1 fusion protein was incubated with purified His tagged TEAD1 from *E. coli* for 4 - 8 h at 4 °C. Then, the protein-bound glutathione-Sepharose 4B beads were washed four times and the proteins were recovered. Finally, the recovered proteins were analyzed by SDS-PAGE.

## Results

### SNHG16 is upregulated in CRC tissues and indicates a poor prognosis in CRC patients

**Figure 1:**
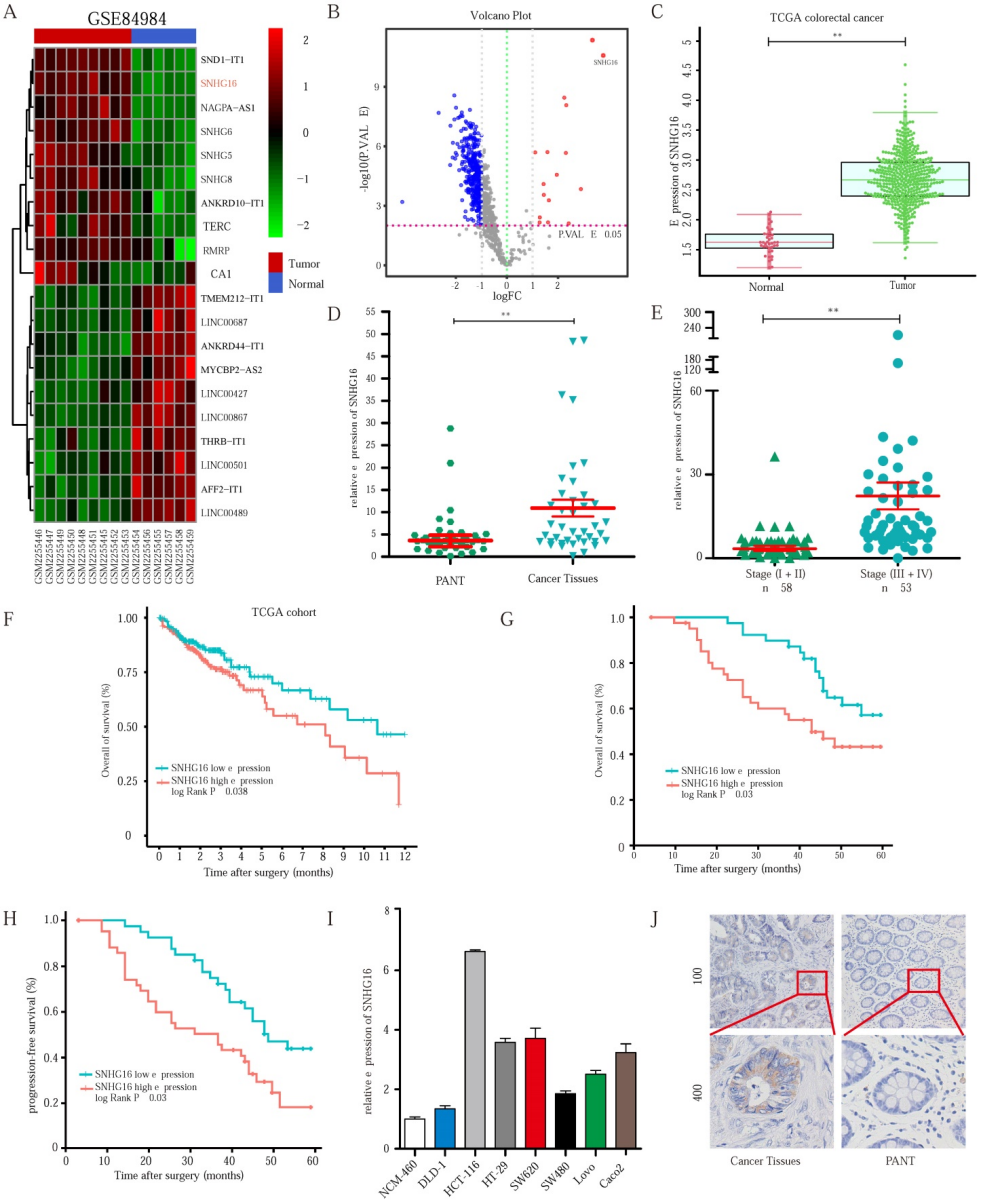
** SNHG16 is upregulated in colorectal cancer tissues and indicates poor prognosis of colorectal cancer patients. A:** Heatmap of most differentially expressed lncRNAs in GSE84984.** B:** Differential expressions of lncRNAs were shown in volcano plots. **C:** SNHG16 expression was validated by qRT-PCR in TCGA datasets, mean **±** SD is shown. **D:** qRT-PCR analyses of SNHG16 expression in CRC tumor tissue (n = 45) and paired non-tumor tissue (n = 45), mean **±** SEM is shown. **E:** qRT-PCR analyses of SNHG16 expression in different clinical stages, mean **±** SEM is shown. **F:** Kaplan-Meier analyses of the correlation between SNHG16 expression level and OS in TCGA cohort. **G-H:** Kaplan-Meier analyses of the correlation between SNHG16 expression and OS or PFS of CRC patients.** I:** Relative expression of SNHG16 in CRC cell lines and colon epithelial cell line.** J:** ISH analyses of SNHG16 expression in cancer tissues and adjacent normal tissues. All representative data are from three independent experiments. Statistical analysis was conducted using Student's t-test. Error bars, SEM. *p < 0.05, **p < 0.01.

To identify the lncRNAs involved in CRC progression, we analyzed the lncRNA expression profiles of CRC in the dataset GSE84984. A series of abnormally expressed lncRNAs in CRC were identified, and SNHG16 was among the most upregulated lncRNAs (Fig. 1A). The top 10 upregulated and downregulated lncRNAs are shown in a heatmap (Fig. 1A). A volcano plot shows the upregulated and downregulated lncRNAs in CRC (Fig. 1B). As mentioned above, lncRNA SNHG16 plays an important role in tumor progression. Thus, lncRNA SNHG16 was selected for further research.

As shown in Fig. 1C and Fig. 1D, SNHG16 expression was significantly upregulated in CRC tissues in the TCGA database and our cohort, and its high expression correlated the advanced tumor-node-metastasis (TNM) stage (Fig. 1E). We then separated 111 CRC tissue samples into two groups based on the median SNHG16 expression level. Subsequently, we found that high SNHG16 expression was positively correlated with lymphovascular invasion (LVI), perineural invasion (PNI), TNM stage, lymph node metastasis, and distant metastasis (Table 1). Further Kaplan-Meier analysis of TCGA database (Fig 1F) and our cohort (Fig. 1G and Fig. 1H) revealed that high SNHG16 expression was associated with poor overall survival (OS) and progression-free survival (PFS) of CRC patients.

Cox regression analysis (Table 2) revealed that SNHG16 expression is an independent prognostic factor that correlated with poor OS (HR = 3.125, 95% CI = 1.145-8.47). Similarly, we found that SNHG16 was significantly upregulated in CRC cell lines (Fig 1I); moreover, we detected the highest expression level of SNHG16 in HCT116 cells and the lowest expression levels of SNHG16 in DLD1 cells; thus, these two cell lines were selected for further research. Additionally, *in situ* hybridization (ISH) showed that SNHG16 was mainly located in the cytoplasm of CRC but not in paired adjacent normal tissue (PANTs) (Fig. 1J). Taken together, these results strongly indicate that SNHG16 is upregulated in CRC and is an independent prognostic factor.

### The ectopic expression of lncRNA SNHG16 affects the proliferation, migration, invasion, and EMT of CRC cells

**Figure 2:**
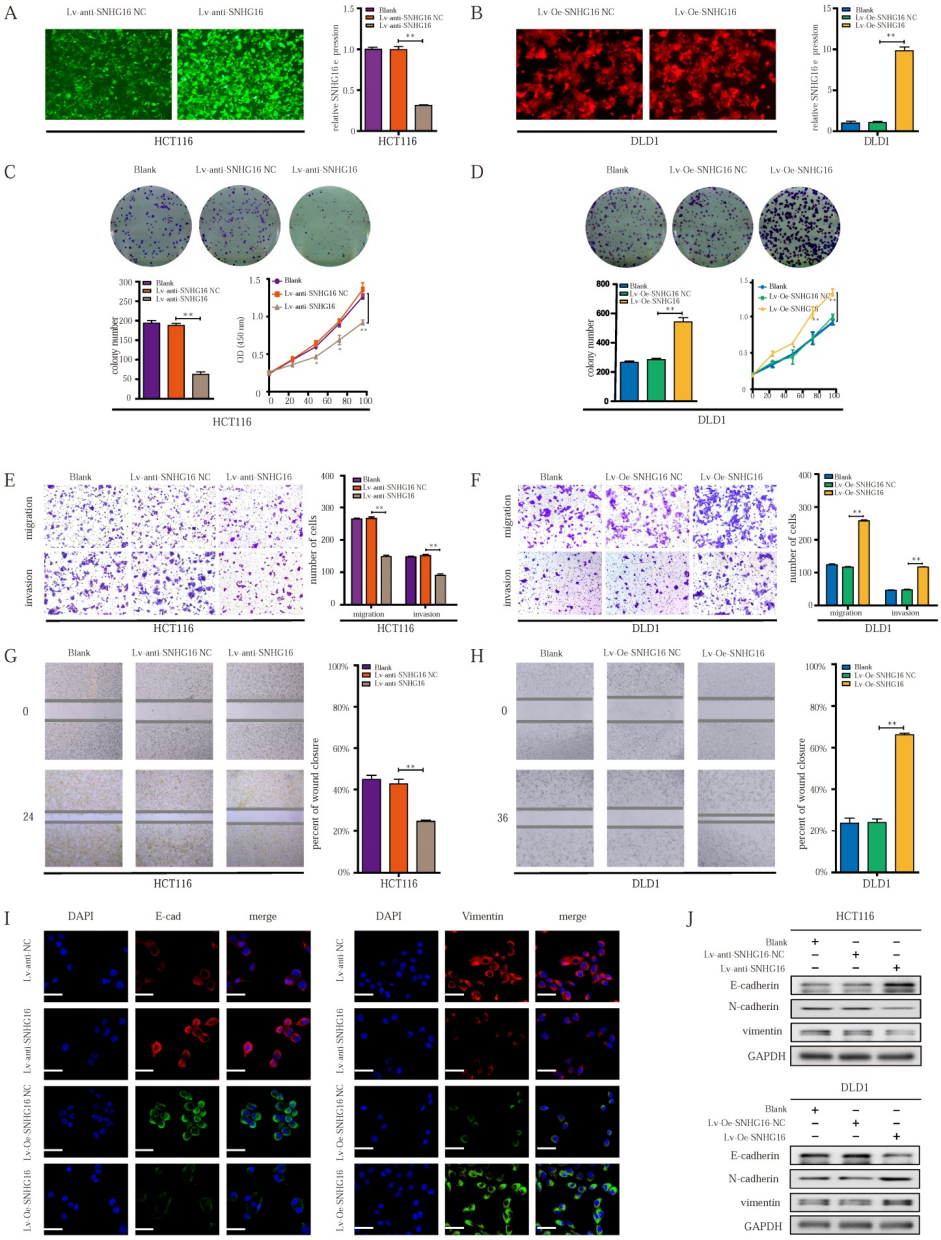
** The ectopic expression of lncRNA-SNHG16 affects proliferation, migration, invasion, and EMT of CRC cells. A:** Micrographs of lentivirus-mediated SNHG16 knockdown in HCT116 and the knockdown effect were validated. **B:** Micrographs of lentivirus-mediated SNHG16 overexpression in DLD1 and the overexpression effect were validated. **C-D:** Colony formation and proliferation assays were performed after SNHG16 knockdown (C) or overexpression (D) in HCT116 or DLD1, respectively. **E-F:** Migration and invasion assays of CRC cell line after SNHG16 knockdown (E) or overexpression (F), respectively. **G-H:** Wound healing assay was performed in CRC cell lines following SNHG16 knockdown or overexpression. **I-J:** EMT-associated markers were detected by immunofluorescence (I) and WB (J) in CRC cell lines with SNHG16 knockdown or overexpression. All representative data are from three independent experiments. Statistical analysis was conducted using one-way ANOVA. Error bars, SEM. *p < 0.05, **p < 0.01.

To investigate the role of SNHG16 in tumor progression, loss- and gain-of-function approaches were applied. First, qRT-PCR was used to identify the most efficient siRNA sequence targeting SNHG16 (Fig. S1A). Then, a lentiviral-based SNHG16 knockdown cell line (Fig. 2A) and a stable SNHG16 overexpression cell line (Fig. 2B), termed Lv-anti-SNHG16 and Lv-Oe-SNHG16 respectively were constructed.

Colony formation and CCK8 assays indicated that cell colony formation and proliferation were inhibited by SNHG16 knockdown (Fig. 2C). In contrast, SNHG16 overexpression promoted the colony formation and proliferation of CRC cells (Fig. 2D). Transwell migration assay, Transwell invasion assay, and wound healing assays showed that SNHG16 knockdown significantly inhibited the migration and invasion of CRC cells (Fig. 2E and Fig. 2G), whereas SNHG16 overexpression promoted the migration and invasion of CRC cells (Fig. 2F and Fig. 2H).

Given the critical role of EMT in CRC cell migration and invasion 51, we investigated whether SNHG16 could induce EMT in CRC cells. WB and immunofluorescence assays revealed the knockdown of SNHG16 significantly reduced the expression levels of vimentin and N-cadherin but increased E-cadherin levels in HCT116 cells (Fig. 2I and Fig. 2J). In contrast, SNHG16 overexpression promoted the EMT process in DLD1 cells (Fig. 2I and Fig. 2J). Protein quantification of WB revealed that the expression of EMT markers significantly differed between different groups (Fig. S1B).

Altogether, these results demonstrate that SNHG16 can regulate CRC cell migration and invasion by affecting EMT process.

### LncRNA SNHG16 facilitates CRC cell proliferation, migration, invasion and EMT in a YAP1-dependent manner

**Figure 3:**
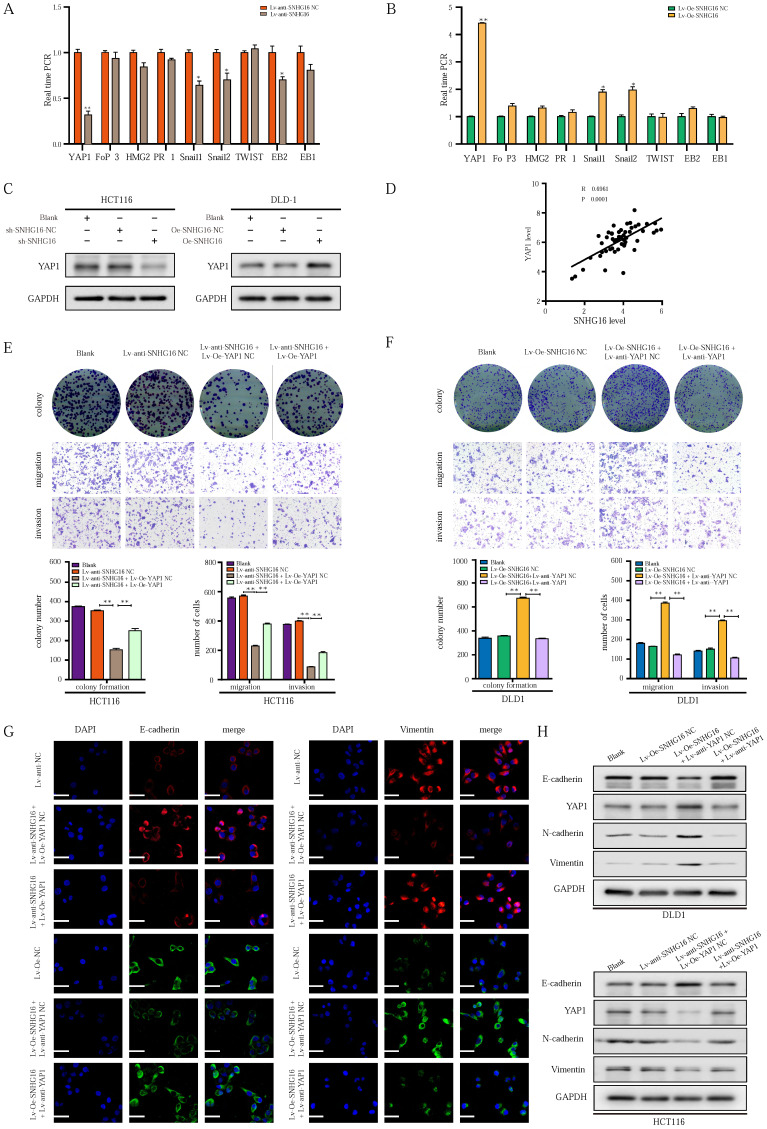
** LncRNA-SNHG16 facilitates CRC cellular proliferation, migration, invasion, and EMT in a YAP1-dependent manner. A-B:** Relative expression levels of representative EMT-related transcription factors were detected following SNHG16 knockdown (A) or overexpression (B), respectively. **C:** YAP1 protein levels were assessed after SNHG16 knockdown or overexpression in CRC cells, respectively. **D:** The correlations between the expression SNHG16 and YAP1 in CRC tissues were analyzed by Pearson's correlation. **E:** Colony formation and Transwell assay were performed to determine the colony formation, migration, and invasion ability of HCT116 co-transfected with Lv-anti-SNHG16 and Lv-Oe-YAP1. **F**: Colony formation and Transwell assay were performed to determine the colony formation, migration, and invasion ability of DLD1 co-transfected with Lv-Oe-SNHG16 and Lv-anti-YAP1. **G**: The expression level of EMT markers in HCT116 (HCT116^ Blank^, HCT116^ Lv-anti-SNHG16-NC^, HCT116^ Lv-anti-SNHG16 + Lv-Oe-YAP1-NC^, HCT116^ Lv-anti-SNHG16 + Lv-Oe-YAP1^) and DLD1 (DLD1^ Blank^, DLD1^ Lv-Oe-SNHG16-NC^, DLD1^ Lv-Oe-SNHG16 + Lv-anti-YAP1 NC^, DLD1^ Lv-Oe-SNHG16 + Lv-anti-YAP1^) was detected by immunofluorescence. **H**: The expression level of EMT markers and YAP1 in HCT116 (HCT116^ Blank^, HCT116^ Lv-anti-SNHG16-NC^, HCT116^ Lv-anti-SNHG16 + Lv-Oe-YAP1-NC^, HCT116^ Lv-anti-SNHG16 + Lv-Oe-YAP1^) and DLD1 (DLD1^ Blank^, DLD1^ Lv-Oe-SNHG16-NC^, DLD1^ Lv-Oe-SNHG16 + Lv-anti-YAP1 NC^, DLD1^ Lv-Oe-SNHG16 + Lv-anti-YAP1^) was detected by WB. All representative data are from three independent experiments. Statistical analysis was conducted using Student's t-test of one-way ANOVA. Error bars, SEM. *p < 0.05, **p < 0.01.

To explore the mechanism by which SNHG16 regulates EMT in CRC cells, we focused on identifying an EMT-related transcription factor 52. As shown in Fig. 3A and 3B, among transcription factors, the expression of YAP1 was altered to the greatest extent in the treatment group. WB analysis (Fig. 3C) and corresponding protein quantification (Fig. S2A) also revealed that SNHG16 could positively regulate the protein level of YAP1, indicating a potential interaction between SNHG16 and YAP1. In addition, the positive association between the expression levels of SNHG16 and YAP1 in CRC tissues suggested that YAP1 is a potential target of SNHG16 (Fig. 3D). Furthermore, we previously demonstrated that the miR-195-5p/YAP1 axis plays a vital role in CRC progression 39. Thus, YAP1 was selected for further research.

To investigate whether the function of SNHG16 in tumor progression is dependent on YAP1, we performed a rescue experiment. SNHG16 knockdown inhibited the colony formation (Fig. 3E), proliferation (Fig. S2B), migration (Fig. 3E and Fig. S2C), and invasion (Fig. 3E) of CRC cells, and YAP1 overexpression rescued the effect of SNHG16 knockdown on tumor progression. Moreover, SNHG16-overexpression promoted the colony formation (Fig. 3F), proliferation (Fig. S2B), migration (Fig. 3F and Fig. S2D), and invasion (Fig. 3F) of DLD1 cells, but these effects were abrogated by YAP1 knockdown. As expected, SNHG16 knockdown induced HCT116 cells to adopt an epithelial phenotype, and YAP1 overexpression caused SNHG16 knockdown cells to revert to a mesenchymal phenotype (Fig. S2E). In contrast, SNHG16-overexpression induced mesenchymal-like morphological features in DLD1. Knockdown of YAP1 caused SNHG16 overexpressing cells to revert to an epithelial phenotype (Fig. S2F). Immunofluorescence and WB assays revealed that YAP1 rescued the effect of SNHG16 on the EMT process (Fig. 3G and Fig. 3H). Protein quantification of WB revealed that the differences between the groups were statistically significant (Fig. S2G). These results reveal that YAP1 is a functional mediator of SNHG16 in CRC.

### MiR-195-5p potently abrogates the effect of the SNHG16/YAP1 axis on tumor progression

**Figure 4:**
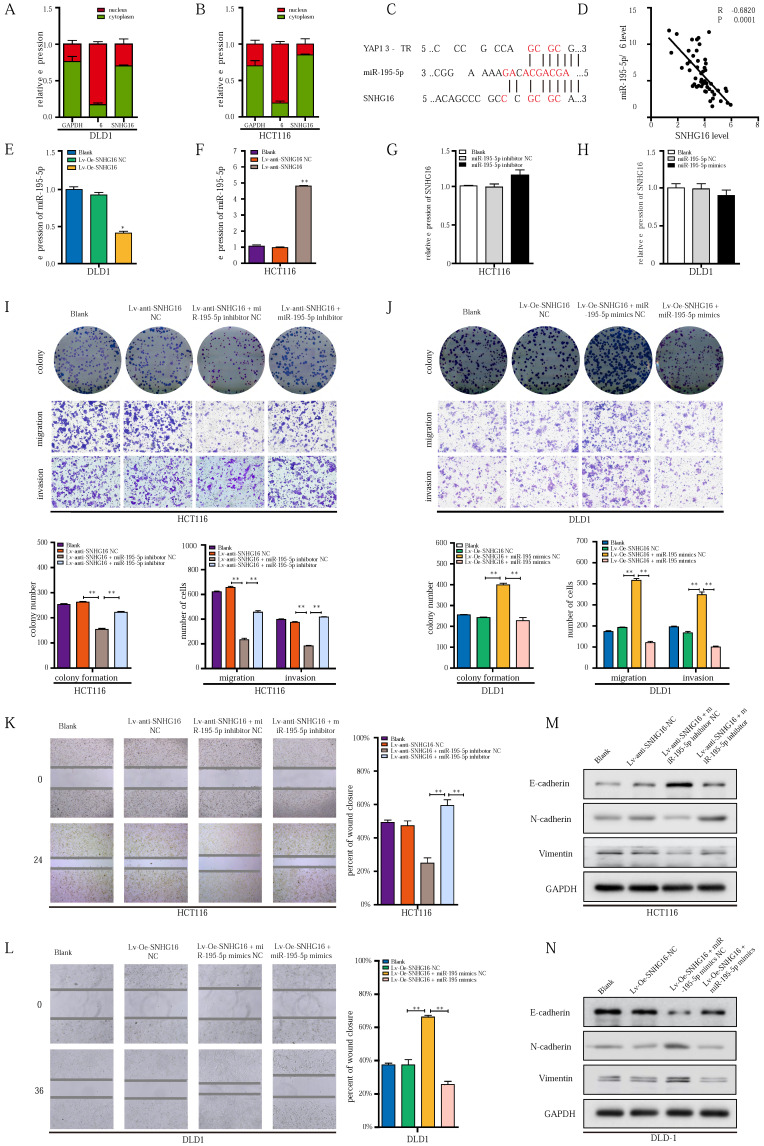
** miR-195-5p could potently abrogate the effect of SNHG16/YAP1 axis on tumor progression. A-B:** The cellular localization of SNHG16 in HCT116 (A) and DLD1 (B) was identified by subcellular fractionation assay.** C:** The putative miR-195-5p binding sites with SNHG16 and YAP1 3′-UTR were shown.** D:** The correlations between SNHG16 and YAP1 expression in CRC tissues were analyzed by Pearson's correlation. **E:** MiR-195-5p expression levels in DLD1 after SNHG16 overexpression. **F**: MiR-195-5p expression levels in HCT116 after SNHG16 overexpression. **G:** SNHG16 expression levels in HCT116 after the miR-195-5p knockdown. **H**: SNHG16 expression levels in DLD1 after miR-195-5p overexpression. **I:** The colony formation, migration, and invasion of HCT116^ Blank^, HCT116^ Lv-anti-SNHG16-NC^, HCT116^ Lv-anti-SNHG16 + miR-195-5p inhibitor NC^, and HCT116^ Lv-anti-SNHG16 + miR-195-5p inhibitor^ was detected by colony formation, and Transwell assay. **J**: The colony formation, migration and invasion of DLD1 (DLD1^ Blank^, DLD1^ Lv-Oe-SNHG16-NC^, DLD1^ Lv-Oe-SNHG16 + miR-195-5p mimics NC^, DLD1^ Lv-Oe-SNHG16 + miR-195-5p mimics^) was detected by colony formation and transwell assay. **K**: Wound healing assays were performed to determine the migration ability of HCT116 co-transfected with Lv-anti-SNHG16 and miR-195-5p inhibitor. **L**: Wound healing assays were performed to determine the migration ability of HCT116 co-transfected with Lv-Oe-SNHG16 and miR-195-5p mimics. **M**: The protein expression level of EMT markers in SNHG16 knockdown-HCT116 cells with or without miR-195-5p inhibitor. **N**: The protein expression level of EMT markers in SNHG16 overexpression-DLD1 cells with or without miR-195-5p mimics. All representative data are from three independent experiments. Statistical analysis was conducted using one-way ANOVA. Error bars, SEM. *p < 0.05, **p < 0.01.

To further investigate how the EMT of CRC cells might be dynamically controlled by the SNHG16/YAP1 axis during tumor metastasis, we focused on the involvement of miRNAs 45. According to the competing endogenous RNA (ceRNA) hypothesis, lncRNAs can function as miRNA sponges to posttranscriptionally regulate target genes in the cytoplasm 45. In our previous research, we demonstrated that miR-195-5p potently inhibited the EMT of CRC cells through YAP1 39. Subcellular distribution assays showed that SNHG16 was mainly located in the cytoplasm of HCT116 and DLD1 cells, indicating potential posttranscriptional regulation (Fig. 4A and Fig. 4B). Through starBase 53 and LncBase Predicted 54, we identified several miRNAs with potential SNHG16 binding sites, including miR-195-5p. Furthermore, we found the same miR-195-5p response elements in SNHG16 and YAP1 (Fig. 4C), which indicated potential interplay among these three factors. Thus, we selected miR-195-5p for further research.

Based on our previous research on the miR-195-5p/YAP1 axis 39; we further investigated the relationship between SNHG16 expression and miR-195-5p. Importantly, SNHG16 expression was significantly negatively correlated with miR-195-5p expression in CRC tissues (two-sided Pearson's correlation, r = -0.6820, p < 0.001) (Fig. 4D). Additionally, SNHG16 negatively regulated the expression of miR-195-5p in DLD1 and HCT116 cells (Fig. 4E and Fig. 4F). However, we did not observe significant changes in SNHG16 expression when we upregulated or downregulated miR-195-5p expression levels (Fig. 4G and Fig. 4H).

Next, we investigated whether the SNHG16/YAP1 axis promotes the migration, invasion, and EMT of CRC cells through miR-195-5p. SNHG16 knockdown inhibited the colony formation (Fig. 4I), proliferation (Fig. S3A), migration (Fig. 4I) and invasion (Fig. 4I) of CRC cells, but, miR-195-5p inhibition clearly rescued the inhibitory effect of SNHG16 knockdown on HCT116 cells. In contrast, miR-195-5p mimics abrogated the enhanced colony formation, proliferation, migration and invasion of SNHG16 overexpressing DLD1 cells (Fig. 4J, Fig. S3B). Then, a wound healing assay further revealed that miR-195-5p could rescue the effect of SNHG16 on migration in CRC cell lines (Fig. 4K-L). Furthermore, WB (Fig. 4M) and corresponding protein quantification (Fig. S3C) indicated that the inhibition of EMT in HCT116 cells with SNHG16 knockdown could be rescued by miR-195-5p inhibitors. Moreover, miR-195-5p mimics significantly reversed the enhancement of EMT in DLD1 cells overexpressing SNHG16 (Fig. 4N and Fig. S3D). Based on the current study and previous research 39, we successfully demonstrated that miR-195-5p could abrogate the effect of the SNHG16/YAP1 axis on tumor progression.

### lncRNA SNHG16 functions as a ceRNA and sponges miR-195-5p through physically binding, further regulating YAP1 expression and facilitating tumor progression

Next, we sought to examine whether SNHG16-mediated miR-195-5p regulation occurs through direct binding with miR-195-5p. Therefore, RNA-binding protein immunoprecipitation (RIP), biotin-avidin pulldown, and luciferase assays were applied in further research.

**Figure 5:**
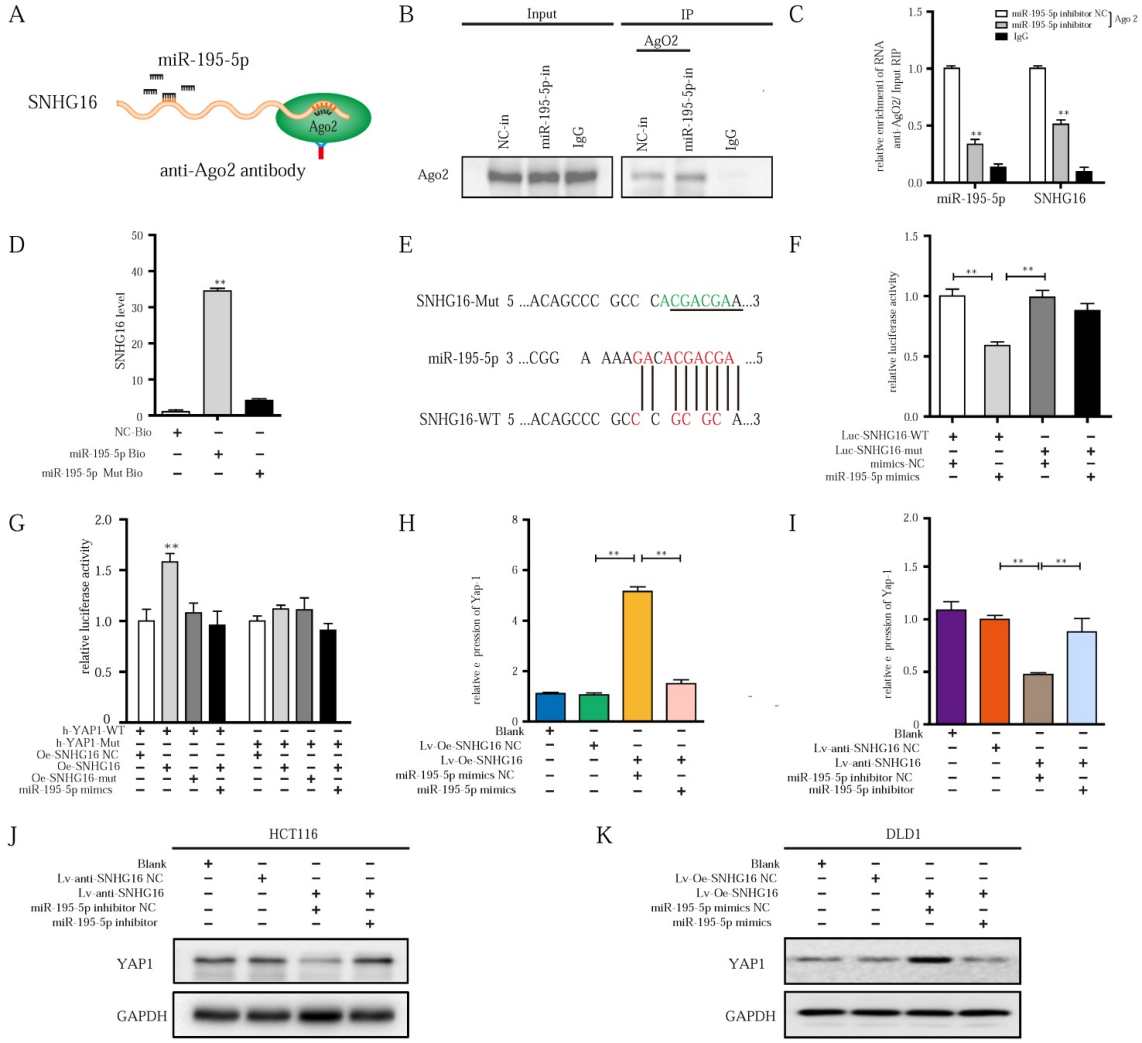
** lncRNA-SNHG16 functions as a ceRNA and sponges miR-195-5p, further regulating YAP1 expression and facilitating tumor progression. A:** Schematic illustration of anti-Ago2 RIP strategy. **B:** Ago2 protein was immunoprecipitated and purified from cell extracts and was further detected by WB. **C:** In the presence of miR-195-5p inhibitor or negative control, the relative expression of SNHG16 and miR-195-5p bound to Ago2 or IgG was measured by RT-qPCR. **D:** Interaction between miR-195-5p and SNHG16 was confirmed by RNA pulldown assay. **E:** The position of the miR-195-5p binding site in SNHG16 is shown. Mutation (underlined) was introduced into SNHG16 to disrupt base-pairing with miR-195-5p seed sequence. **F:** A dual-luciferase reporter assay was performed in 293T to reveal the binding of miR-195-5p to SNHG16. **G:** Luciferase assay among SNHG16 (SNHG16-WT and SNHG16-mut), miR-195-5p, and dual-luciferase vector YAP1 (WT and mut) were performed in 293T to confirm the interplay of these three factors. **H:** The relative expression of YAP1 mRNA in SNHG16-overexpressed-DLD1 cells with or without miR-195-5p mimics. **I:** The relative expression of YAP1 mRNA in SNHG16 knockdown-HCT116 cells with or without miR-195-5p inhibitor. **J:** The YAP1 protein expression in SNHG16 knockdown-HCT116 cells with or without miR-195-5p inhibitor. **K:** The YAP1 protein expression in SNHG16 overexpressed-DLD1 cells with or without miR-195-5p mimics. All representative data are from three independent experiments. Statistical analysis was conducted using one-way ANOVA. Error bars, SEM. *p < 0.05, **p < 0.01.

Previous studies verify that miRNAs are present in the form of miRNA ribonucleoprotein complexes (miRNPs) that contain Ago2, which is the core component of RNA-induced silencing complexes (RISCs) 55. To validate whether miR-195-5p associates with SNHG16, we performed RIP assays with anti-Ago2 antibody (IgG as blank control) on HCT116^ miR-195-5p inhibitor NC^ or HCT116^ miR-195-5p inhibitor^ (Fig. 5A). As shown in Fig. 5B, the Ago2 protein was successfully immunoprecipitated from HCT116^ miR-195-5p inhibitor^ and HCT116^ miR-195-5p inhibitor NC^ with the Ago2 antibody. RIP verified that SNHG16 was detected in HCT116^ miR-195-5p inhibitor NC^ group, and SNHG16 expression was drastically reduced in Ago2 complexes purified from the HCT116^ miR-195-5p inhibitor^ group, indicating that SNHG16 was in the miR-195-5p RISCs (Fig. 5C) and miR-195-5p bound with SNHG16. Additionally, sequence-specific binding between miR-195-5p and SNHG16 was further validated by a biotin-avidin miR-195-5p pulldown system. As Fig. 5D shown, the expression level of SNHG16 in the miR-195-5p-Bio group was approximately 30 times higher than that in the control group, and the introduction of mutations (miR-195-5p Mut) disrupted base pairing between SNHG16 and miR-195-5p. RNA pull-down assays suggested that miR-195-5p interacts with SNHG16 in a sequence-specific manner. Consistent with our results, luciferase reporter assays revealed that miR-195-5p significantly inhibited the luciferase activity of the SNHG16-wild type (WT) construct but not that of the SNHG16-mutant (mut) construct (Fig. 5E-F). Collectively, these results revealed that the binding of SNHG16 to miR-195-5p is sequence-specific. Based on the data in Fig. 4 and Fig. 5, we ultimately demonstrated that SNHG16 regulates the expression of miR-195-5p through physically binding.

As we previously demonstrated 39, the upregulation of miR-195-5p suppresses the progression of CRC cells by targeting the YAP1 mRNA 3'- untranslated region (UTR). Through starBase, we also found many identical miR-195-5p response elements in SNHG16 and YAP1 (Fig. 4C). Thus, we speculated that SNHG16 can regulate the mRNA stability of YAP1 through miR-195-5p. Then, a luciferase assay of SNHG16 (WT and mut), miR-195-5p, and 3'-UTR-YAP1 (Luc-YAP1-wt and Luc-YAP1-mut) was performed. Compared with SNHG16-mut, SNHG16-WT enhanced the luciferase activity of YAP1-WT, while miR-195-5p inhibited the luciferase activity of YAP1-WT. Furthermore, the inhibition of luciferase activity in the miR-195-5p group on YAP1-WT was rescued by SNHG16 (Fig. 5G). We did not find a significant difference when we conducted the same experiment with YAP1-mut (Fig. 5G). Analysis of these three factors with luciferase assays revealed that the overexpression of SNHG16 could prevent miR-195-5p from targeting YAP1. Through WB and qRT-PCR analysis of a CRC cell line, we also found that SNHG16 could positively regulate the mRNA and protein expression of YAP1. Furthermore, miR-195-5p rescued the SNHG16-mediated regulation of YAP1 (Fig. 5H-K). Protein quantification of WB revealed that the differences between different groups were statistically significant (Fig. S4A and Fig. S4B).

Altogether, these findings indicate that SNHG16 can function as a miRNA sponge to directly bind and sequester endogenous miR-195-5p, thereby preventing it from inhibiting YAP1 expression and affecting tumor progression.

### YAP1 combines with TEAD1 to form a complex that binds the promoter region of SNHG16 and activates SNHG16 transcription

**Figure 6:**
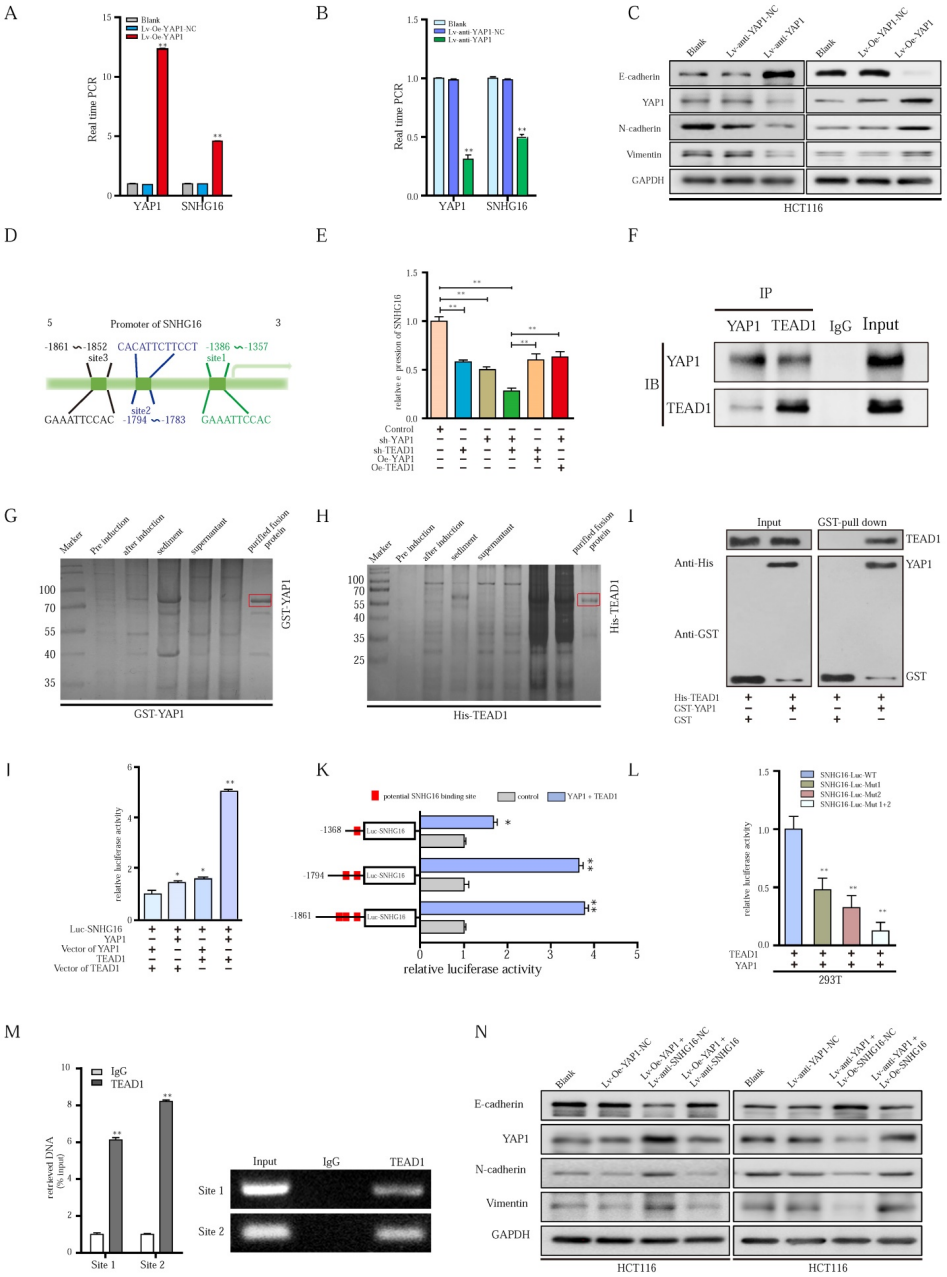
** YAP1 could combine with TEAD1, forming a complex that binds to the promoter region of SNHG16 and activates its transcription. A-B:** Relative expression of SNHG16 was detected following YAP1 overexpression (A) or knockdown (B). **C:** The protein expression levels of EMT markers following YAP1 knockdown or overexpression.** D:** Schematic diagram showing the human SNHG16 upstream promoter region (top), including the predicted TEAD1-binding regions. **E:** Expression levels of SNHG16 were detected when we changed the level of either YAP1 or TEAD1. **F**: Co-IP experiments were used to detect the interplay between YAP1 and TEAD1. **G:** The GST-tagged YAP1 was successfully induced and purified from Escherichia coli. **H:** The His-tagged TEAD1 was successfully induced and purified from Escherichia coli. **I:** GST pull down assay between YAP1 and TEAD1. **J:** A dual-luciferase reporter assay driven by the SNHG16 promoter was co-transfected in the presence or absence of YAP1 or TEAD1. **K:** Selective mutation analyses to detect YAP1/TEAD1-complex-responsive-regions in the SNHG16 promoter in 293T.** L:** Dual-luciferase reporter assay was performed to detect YAP1/TEAD1-complex-responsive-regions in the SNHG16 promoter.** M:** ChIP assay was performed to detect the binding site of TEAD1 to the SNHG16 promoter, including CHIP1 and CHIP2 in HCT116 cells. Input, 10% of total lysate. **N:** The protein expression of E-cadherin, N-cadherin, vimentin, and YAP1 in eight different groups were detected by WB. All representative data are from three independent experiments. Statistical analysis was conducted using one-way ANOVA or Student's t-test. Error bars, SEM. *p < 0.05, **p < 0.01.

Since YAP1 functions as an important transcriptional coactivator, the positive correlation between SNHG16 and YAP1 expression drew us to investigate the regulatory role of YAP1 in SNHG16 transcription. Then, we constructed lentivirus-mediated HCT116 cell lines in which YAP1 were stably overexpressed or knocked down, termed Lv-Oe-YAP1 and Lv-anti-YAP1, respectively. YAP1 overexpression significantly promoted SNHG16 expression (Fig. 6A), whereas YAP1 knockdown potently suppressed the expression level of SNHG16 (Fig. 6B). Furthermore, knockdown of YAP1 inhibited the colony formation (Fig. S5A), migration (Fig. S5C and Fig. S5E), invasion (Fig. S5C), and EMT (Fig. 6C and Fig. S5G) of CRC cell lines. In addition, we observed increased tumor progression in the Lv-Oe-YAP1 group (Fig. S5B, Fig. S5D, Fig. S5F, Fig. 6C and Fig. S5H).

Although we have demonstrated the important role of YAP1 in SNHG16 expression and tumor progression, the mechanism of the YAP1-lncRNA interaction remains largely unknown. Based on existing research, YAP1 cannot directly bind with DNA alone. However, YAP1 can combine with transcription factors to regulate the transcription of downstream genes 56. Among these transcription factors, TEAD 1-4 are some of the most common binding molecules 57, 58. Once bound to TEAD, YAP1 forms a YAP1/TEAD complex to initiate downstream gene transcription 56, 58. We employed JASPAR (http://jaspar.genereg.net) and TRANSFAC (http://gene-regulation.com), and we did not find possible YAP1 binding sites within 2000 bp upstream of the transcriptional start site of SNHG16. Among these transcription factors, only TEAD1 had potential binding sites for the SNHG16 promoter (Fig. 6D). Thus, TEAD1 was selected for further research. Further study showed that the knockdown of YAP1 or TEAD1 reduced SNHG16 expression (Fig. 6E). And evidently, the inhibitory effect on the expression of SNHG16 was clearly most significant when we simultaneously knocked down both YAP1 and TEAD1 (Fig. 6E). To investigate whether YAP1 and TEAD1 act synergistically, we overexpressed TEAD1 in the Lv-anti-YAP1 cell line and did not find a significant change in the expression of SNHG16 (Fig 6E). Similarly, when we overexpressed YAP1 after TEAD1 knockdown, the expression level of SNHG16 was not restored (Fig. 6E). To further verify the cross-talk between TEAD1 and YAP1, coimmunoprecipitation (Co-IP) analysis was applied. Co-IP confirmed that YAP1 could directly or indirectly interact with TEAD1 in HCT116 cells (Fig. 6F). To further investigate whether YAP1 interacts with TEAD1 through direct binding, a GST pulldown assay was performed. After the expression of GST-tagged YAP1 (Fig. 6G) and His-tagged TEAD1 (Fig. 6H) were successfully induced and the protein were purified from Escherichia coli, we incubated the two proteins at 4 °C for 4-8 h. Finally, WB was performed to examine the expression of these two proteins. GST pulldown further demonstrated the direct interplay between YAP1 and TEAD1 (Fig. 6I). Collectively, these experiments confirm that YAP1 can directly interact with TEAD1 and form a complex that regulates the expression of SNHG16. However, whether YAP1/TEAD1 binds the promoter of SNHG16 remains largely unclear.

The interplay between the YAP1/TEAD1 complex and the SNHG16 promoter was further assessed. Significantly, dual-reporter luciferase assays showed that overexpression of either YAP1 or TEAD1 in HCT116 cells stimulated promoter activity of SNHG16 (Fig. 6J). The increase in promoter activity was clearly most significant when we simultaneously overexpressed YAP1 and TEAD1 (Fig. 6J). To verify the binding of SNHG16 and TEAD1, we generated a series of 5' deletion constructs of the SNHG16 promoter. Based on the results of a luciferase assay, the regulatory region between -1794 and -1357 is responsible for YAP1/TEAD1-mediated promoter regulation (Fig. 6K). As shown in Fig 6I, two binding sites are located in this region. Thus, luciferase assays were further performed. YAP1/TEAD1 failed to stimulate mutants of both predicted sites in the promoter region of SNHG16 in 293T cells, while it did affect the WT construct (Fig. 6L). Furthermore, in the chromatin immunoprecipitation (ChIP) assay, we designed two primer sets, one that contained site 1 and one that contained site 2. Then, we used the primers and purified DNA from the ChIP assay to amplify part of the promoter region. The ChIP assay revealed that TEAD1 directly bound both site 1 and site 2 of the SNHG16 promoter in HCT116 cells (Fig. 6M).

The results indicate that YAP1 can directly bind TEAD1 to form a complex, which could, in turn, binds the two sites in the promoter of SNHG16 and regulate SNHG16 transcription. During this process, YAP1 is indispensable for the function of TEAD1, and these two proteins act synergistically.

### Effect of the YAP1-SNHG16 positive feedback loop on tumor progression

We demonstrated that SNHG16 was a direct target of the YAP1/TEAD1 complex and that positive feedback regulation existed between SNHG16 and YAP1. To investigate the role of the YAP1/TEAD1-SNHG16 positive feedback loop in tumor progression, subsequent experiments were performed. Colony formation, CCK8, Transwell, and wound healing assays revealed that YAP1 overexpression significantly promoted tumor progression, whereas the promotive effect on HCT116 was impaired by simultaneous knockdown of SNHG16 (Fig. S6A). Similarly, SNHG16 overexpression rescued the impaired proliferation, migration and invasion of the Lv-anti-YAP1 stable cell line (Fig. S6B). In addition, SNHG16 rescued the effect of YAP1 on CRC EMT (Fig. 6N, Fig. S6C and Fig. S6D).

### Alteration of lncRNA SNHG16 expression influences tumor growth, EMT, ^M^CTC generation, and metastasis *in vivo*

**Figure 7:**
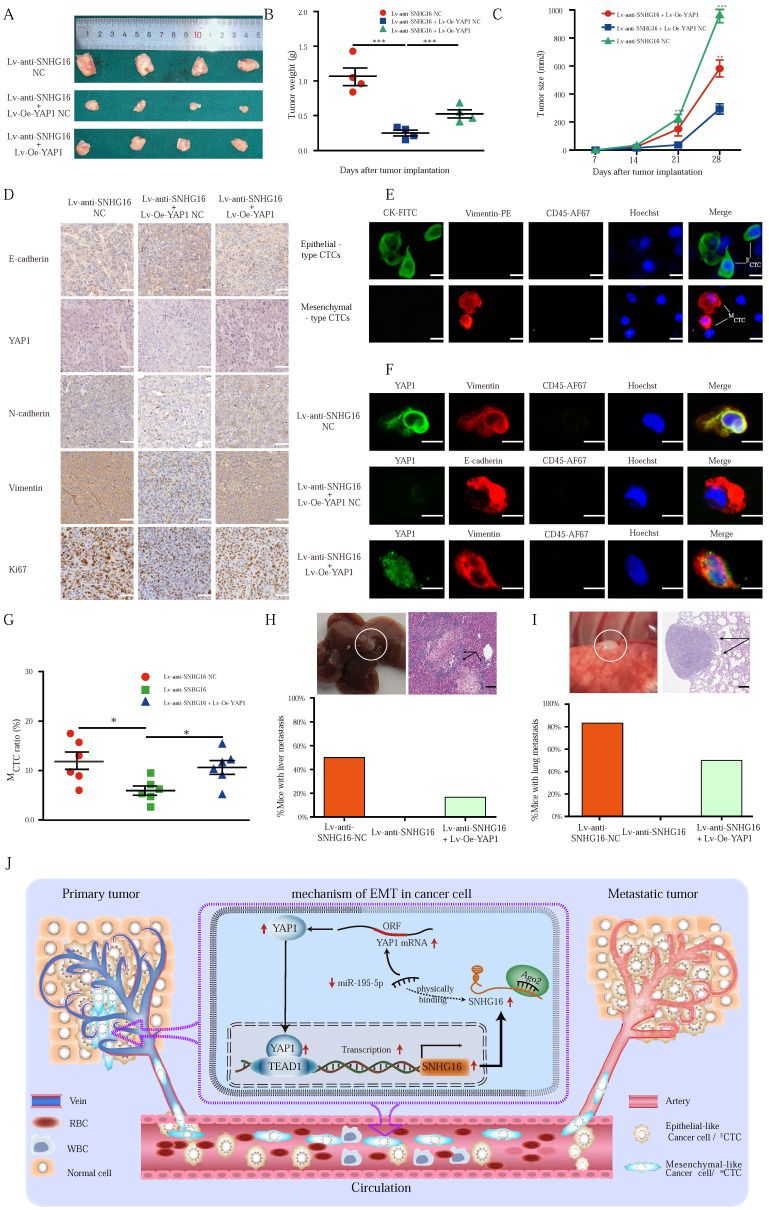
** The alteration of SNHG16 expression influenced the CRC tumorigenesis and CTC generation *in vivo*. A-C**: The morphological characteristics (A), weight (B), and size (C) of tumor xenograft in different group (HCT116^ Lv-anti-SNHG16-NC^, HCT116^ Lv-anti-SNHG16 + Lv-Oe-YAP1-NC^, HCT116^ Lv-anti-SNHG16 + Lv-Oe-YAP1^) injected nude mice. Error bars, SEM. **D:** IHC analyzed the expression of E-cadherin, Vimentin, N-cadherin, Ki67, and YAP1 in different group (HCT116^ Lv-anti-SNHG16-NC^, HCT116^ Lv-anti-SNHG16 + Lv-Oe-YAP1-NC^, HCT116^ Lv-anti-SNHG16 + Lv-Oe-YAP1^). Scale bar, 50 µm. **E**: Representative images of CTC isolated from two mice, respectively. Epithelial-like CTC (^E^CTC) means CTC is accompanied by overexpression of CK. Mesenchymal-like CTC (^M^CTC) means CTC is accompanied by overexpression of vimentin. Scale bar, 10 µm. **F**: The expression of YAP1 and EMT marker on CTCs that isolated from different group (HCT116^ Lv-anti-SNHG16-NC^, HCT116^ Lv-anti-SNHG16 + Lv-Oe-YAP1-NC^, HCT116^ Lv-anti-SNHG16 + Lv-Oe-YAP1^). Scale bar, 10 µm. **G:** The ^M^CTC ratio of mouse blood collected from different group (HCT116^ Lv-anti-SNHG16-NC^, HCT116^ Lv-anti-SNHG16 + Lv-Oe-YAP1-NC^, HCT116^ Lv-anti-SNHG16 + Lv-Oe-YAP1^) Error bars, SEM. **H-I**: Representative images of metastatic lesions in the liver of mice in the HCT116/Lv-anti-SNHG16 NC group, and representative images of metastatic lesions in the lung of mice in the HCT116^ Lv-anti-SNHG16 + Lv-Oe-YAP1^ group. Representative hematoxylin and eosin-stained sections of metastatic nodules in the liver and lung are shown. Scale bar, 100 µm. **J:** A schematic diagram illustrated the mechanism by which LncRNA SNHG16 promoted CRC progression and liver metastasis. All representative data are from three independent experiments. Statistical analysis was conducted using one-way ANOVA. Error bars, SEM. *p < 0.05, **p < 0.01.

To verify the above results *in vivo*, a nude mouse xenograft experiment was performed. The stable cell lines HCT116^ Lv-anti-SNHG16-NC^, HCT116^ Lv-anti-SNHG16 + Lv-Oe-YAP1 NC^, and HCT116^ Lv-anti-SNHG16 + Lv-Oe-YAP1^ were individually injected into the flanks of nude mice separately. During tumor growth period, both the tumor weight and tumor volume were measured. Evidently, knockdown of SNHG16 clearly inhibited the growth of the tumors. Furthermore, the inhibitory effect of SNHG16 on tumor growth was rescued by YAP1 overexpression (Fig. 7A, B and C).

IHC was performed to detect the expression of YAP1 and EMT-related factors in xenograft tumors (Fig. 7D), and the expression of the proteins assessed by IHC was also quantified by IOD (Fig. S7). Notably, Ki67 staining was significantly decreased in the Lv-anti-SNHG16 group, indicating inhibited proliferation in the tumors. Moreover, overexpression of YAP1 significantly attenuated the inhibition of Ki67 expression (Fig. 7D). Importantly, IHC staining confirmed the *in vitro* results showing that SNHG16 could regulate the expression of YAP1 and the EMT process. Moreover, YAP1 overexpression rescued SNHG16 knockdown-mediated inhibition of EMT (Fig. 7D). Quantification of the IHC results indicated that the differences in the expression of the EMT markers, YAP1, and Ki67 were statistically significant (Fig. S7).

Through the EMT process, cancer cells can shed from tumors and invade the blood, thereby forming CTCs and subsequent tumor metastases. Thus, we detected CTCs in mouse blood (Fig. 7E). We then detected representative markers of EMT on CTCs. As shown in Fig. 7F, SNHG16 regulated the expression of YAP1 and EMT markers, and YAP1 overexpression rescued SNHG16 knockdown-mediated inhibition of EMT marker expression. Obviously, Lv-anti-SNHG16 significantly decreased the rate of ^M^CTC generation, compared with that in a negative control group (Fig. 7G); moreover, YAP1 overexpression rescued SNHG16 knockdown-mediated inhibition of ^M^CTC generation (Fig. 7G).

Three stable cell lines HCT116^ Lv-anti-SNHG16 NC^, HCT116^ Lv-anti-SNHG16 + Lv-Oe-YAP1 NC^, and HCT116^ Lv-anti-SNHG16 + Lv-Oe-YAP1^ were separately injected into the tail vein of mice to confirm the effect of SNHG16 on tumor metastasis. Representative images of CRC liver metastasis and lung metastasis are presented in Fig. 7H and 7I, respectively. Further analysis revealed that the knockdown of SNHG16 significantly inhibited tumor metastasis. In addition, the overexpression of YAP1 rescued the inhibitory effect of SNHG16 knockdown on tumor metastasis (Fig. 7H and 7I). Collectively, these results demonstrate that SNHG16 can promote tumor growth, metastasis, and ^M^CTC generation and that YAP1 can rescue the effect of SNHG16 on tumor progression.

Our mechanistic findings are summarized in a schematic diagram in Fig. 7I. In summary, our study demonstrates the presence of a positive feedback loop between lncRNA SNHG16 and the YAP1/TEAD1 complex. Mechanistically, SNHG16 can act as a miRNA sponge to sequester miR-195-5p on Ago2, thereby protecting YAP1 from repression. Moreover, YAP1 can combine with TEAD1 to form a YAP1/TEAD1 complex, which can bind the SNHG16 promoter to regulate its transcription. By means of this positive feedback loop, SNHG16 promotes CRC migration, invasion, ^M^CTC generation, lung metastasis, and liver metastasis.

## Discussion

In the present study, we propose a novel function for SNHG16 in CRC progression. Generally, lncRNA SNHG16 can act as a miRNA sponge to sequester miR-195-5p on Ago2, thereby protecting YAP1 from repression. Moreover, YAP1 can combine with TEAD1 to form a YAP1/TEAD1 complex, which in turn binds with SNHG16 promoter to regulate SNHG16 transcription. Furthermore, high expression of SNHG16 is associated with invasion, EMT, metastasis and poor prognosis in CRC cancer patients. All these data demonstrated that SNHG16 has oncogenic activity in CRC.

To provide further insights into the role of SNHG16 in CRC EMT and liver metastasis, we assessed the gene expression of EMT transcription factors after SNHG16 overexpression or knockdown. When we changed the expression level of SNHG16, YAP1, which was identified as an important EMT transcription factor in our recent research 39 and previously published articles 59, 60, changed to the greatest extent among EMT-related factors. Subsequently, we demonstrated that YAP1 rescued the SNHG16-mediated effect on CRC progression *in vitro*. According to recent research, CTCs that are shed from the primary tumor can gain mesenchymal traits via EMT with a consequent increase in their invasive ability and metastatic ability 61. Qi et al. 62 and our laboratory 25 also demonstrated that ^M^CTCs are especially important for tumor recurrence and metastasis. Our results revealed that the ^M^CTC ratio and liver metastasis was significantly decreased after SNHG16 knockdown. Our results indicate that, SNHG16 can regulate the EMT state of CTCs, thus facilitating the generation of ^M^CTC and liver metastasis of CRC. Finally, we demonstrate that YAP1 can rescue the effect of SNHG16 on ^M^CTC generation and the liver metastasis of CRC. Overall, our research has verified that SNHG16/YAP1 axis can mediate CTCs EMT thus facilitating the liver metastasis of CRC for the first time. However, the mechanism of SNHG16 activates YAP1 requires further investigation.

In our previous research, YAP1 was identified as a target gene of miR-195-5p in CRC 39. In a newly described regulatory mechanism, lncRNA can influence posttranscriptional regulation by competing for shared miRNA response elements 45. Interestingly, inverse regulation between SNHG16 and miR-195-5p suggested that miR-195-5p is a potential target gene of SNHG16. A Luciferase assay showed that miR-195-5p binds SNHG16 in a sequence specific manner. What's more, this finding was further confirmed by anti-Ago2 RIP and biotin-avidin labeled miR-195-5p pulldown. Finally, we found that miR-195-5p rescued the effect of SNHG16 on YAP1 expression and the luciferase activity of the YAP1 3'-UTR. For the first time, we have demonstrated that SNHG16 acts as a miRNA sponge to sequester miR-195-5p on Ago2, thereby protecting YAP1 from repression. Collectively, these findings indicate that the crosstalk between RNAs (e.g., lncRNAs and mRNAs) facilitates the formation of a large-scale regulatory network involving different pathways. Thus, ceRNA networks may pave a new path for tumor therapeutic regimens.

The positive regulation of YAP1, an important transcriptional coactivator, by SNHG16 led us to investigate the role of YAP1 in SNHG16 transcription. We have demonstrated for the first time that YAP1 can activate the transcription of SNHG16. However, the molecular mechanism underlying the YAP1-lncRNA interaction remains unknown. According to previous research, YAP1 cannot regulate transcription alone but does so in combination with downstream transcription factors 56, 58, mainly TEAD 1-4 63. Once bound to TEAD, YAP1 relies on the DNA binding domain of TEAD to initiate downstream gene transcription 56, 58, 64. In this study, we identified TEAD1 as a potential downstream transcription factor via UCSC, JASPAR, and TRANSFAC. We then found that only when YAP1 and TEAD1 act in synergy could they regulate the transcription of SNHG16. Then, Co-IP and GST pulldown assay identified the direct interaction between YAP1 and TEAD1. Unsurprisingly, dual-luciferase and ChIP assays revealed that YAP1/TEAD1 could directly bind to two sites in the SNHG16 promoter regulatory region between -1794 and -1367, and thus activating the transcription of SNHG16. Finally, we revealed that YAP1 significantly promoted tumor progression and that SNHG16 rescued the effect of YAP1 on tumor progression. Therefore, SNHG16 and YAP1 form a positive feedback loop to regulate tumor progression.

## Conclusion

In summary, our study demonstrates the presence of a positive feedback loop between lncRNA SNHG16 and the YAP1/TEAD1 complex. Mechanistically, SNHG16 can act as a miRNA sponge to sequester miR-195-5p on Ago2, thereby protecting YAP1 from repression. Moreover, YAP1 can combine with TEAD1 to form a YAP1/TEAD1 complex, which can bind the SNHG16 promoter to regulate SNHG16 transcription. By means of this positive feedback loop, SNHG16 promotes CRC migration, invasion, MCTC generation, lung metastasis, and liver metastasis.

## Supplementary Material

Supplementary figures and tables.

## Figures and Tables

**Table 1:** Correlation between the expression of SNHG16 and clinicopathologic characteristics in CRC

Parameters	n	SNHG16 expression		p
		Low	High	
**Gender**				
Male	63	30	33	0.784
Female	48	25	23	
**Age, years**				
<60	48	27	21	0.298
>60	63	28	35	
**Tumor site**				
<4	58	31	27	0.503
>4	53	24	29	
**Tumor size, cm**				
Colon	56	26	30	0.636
Rectal	55	29	26	
**Tumor differentiation**				
Moderate/well	81	42	39	0.523
Poor	30	13	17	
**LVI**				
Absence	59	38	21	**0.002**
Presence	52	17	35	
**PNI**				
Presence	56	18	38	**0.001**
Absence	55	37	18	
**TI**				
T1-2	15	11	4	0.056
T3-4	96	44	52	
**LNM**				
N0-1	86	52	34	**0.003**
N2-N3	25	3	22	
**TNM stage**				
I/II	57	48	9	**0.001**
III/IV	54	7	47	
**CA199**				
<27	77	42	35	0.15
>27	34	13	21	
**CEA**				
<5	59	35	24	**0.037**
>5	52	20	32	
**M**				
No metastasis	101	55	46	**0.001**
Metastasis	10	0	10	
Overall	111	55	56	

**Table 2:** Univariate and multivariate analyses of clinicopathologic parameters associated with progression-free survival and overall survival

Parameters	Overall survival						Progression-free survival					
	Univariate analysis			Multivariate analysis			Univariate analysis			Multivariate analysis		
	HR	95%CI	P	HR	95%CI	P	HR	95%CI	P	HR	95%CI	P
**Gender** Female vs Male	1.19	0.625-2.69	0.756				1.076	0.624, 1.856	0.792			
**CA19-9** <27 vs >27	0.806	0.404-1.607	0.54				0.666	0.377, 1.177	0.162			
CEA (low vs high)	0.731	0.382-1.397	0.343				0.872	0.505, 1.507	0.624			
PNI (low vs high)	0.738	0.387-1.408	0.229				0.625	0.363, 1.074	0.259			
LNM (low vs high)	0.225	0.115-0.443	**0**	0.51	0.224-1.143	**0.06**	0.32	0.173, 0.591	**0.001**	0.819	0.399-1.681	0.586
Tumor size (<4 cm vs >4 cm)	0.9	0.472-1.715	0.748				0.587	0.338, 1.018	0.058			
Tumor site (rectal vs colon)	1.683	0.879- 3.224	0.135				1.657	0.962, 2.854	0.069			
Age (<60 vs >60)	0.702	0.44-1.344	0.286				1.076	0.624, 1.856	0.792			
TI (T1-2 vs T3-4)	0.443	0.157-1.254	0.125				0.31	0.123, 0.783	0.013	0.417	0.163-1.064	0.067
**Tumor differentiation**poor/well vs moderate	1.18	0.583-2.390	0.645				1.243	0.691, 2.236	0.467			
**Metastasis** absence vs presence	0.031	0.011-0.086	**0.001**	0.03	0.009-0.102	**0.01**	0.02	0.005,0.077	**0.000**	0.028	0.007-0.113	**0.000**
**TNM stage^a^** I-II vs III-IV	0.226	0.110-0.463	**0.002**	0.19	0.064-0.564	**0.01**	0.26	0.142,0.458	**0.000**	0.352	0.188-0.658	**0.001**
**SNHG16** Low vs High	0.493	0.255-0.953	**0.045**	0.32	0.118-0.873	**0.03**	0.41	0.236,0.726	**0.002**	0.535	0.226-1.269	**0.156**
**LVI** absence vs presence	0.327	0.169-0.633	**0.003**	0.44	0.210-0.937	**0.00**	0.319	0.184,0.555	**0.000**	0.625	0.332-1.176	**0.145**

**Notes**: ^a^The 8th edition of the AJCC Cancer Staging Manual; Boldface indicates P < 0.05; **Abbreviations**: LVI lymphovascular invasion, PNI perineural invasion, TI tumor invasion, LNM lymph node metastasis, TNM tumor-node-metastasis, CA19-9 carbohydrate antigen 19-9, CEA carcinoembryonic antigen.

## Abbreviations

3′-UTR: 3'-untranslated region
western blotting: WB
CRC: Colorectal cancer
CTC: circulating tumor cell
^E^CTC: epithelial-like circulating tumor cell
^M^CTC: mesenchymal-like circulating tumor cell
EMT: Epithelial-mesenchymal transition
SNHG16: Long noncoding RNA SNHG16
miR: MicroRNA/miRNA
miR-195-5p: Hsa-microRNA-195-5p
NC: Negative control
PANT: Paired adjacent normal tissue
qRT-PCR: Quantitative reverse transcription polymerase chain reaction
SD: Standard deviation
YAP1: Yes-associated protein 1
Lv-Oe-SNHG16: lentivirus mediated overexpression of SNHG16
Lv-Oe-SNHG16 NC: nonspecific Lentiviral vectors of SNHG16 overexpression
Lv-Oe-YAP1: lentivirus mediated overexpression of YAP1
Lv-Oe-YAP1 NC: nonspecific Lentiviral vectors of YAP1 overexpression
LVI: lymphovascular invasion
PNI: perineural invasion
TI: tumor invasion
LNM: lymph node metastasis
TNM: tumor-node-metastasis
CA19-9: carbohydrate antigen 19-9
CEA: carcinoembryonic antigen
IHC: immunohistochemistry
