# Supplementary material for: SNHG16 upregulation-induced positive feedback loop with YAP1/TEAD1 complex in Colorectal Cancer cell lines facilitates liver metastasis of colorectal cancer by modulating CTCs epithelial-mesenchymal transition
Source: Int J Biol Sci. 2022 Aug 16;18(14):5291–308. doi: 10.7150/ijbs.73438 (PMC9461660; doi:10.7150/ijbs.73438)
Supplement: Supplementary file 1 — Supplementary figures and tables. [file ijbsv18p5291s1.pdf]

supplementary material and method

METHOD AND MATERIAL

Dataset's analysis

The CRC-related RNA-sequencing dataset GSE84984 was obtained from the Gene Expression Omnibus (GEO) database. The GSE84984 dataset contains nine tumor tissues and six normal tissues. Then, we filtered the background noise of the gene expression profile and then performed the data analysis using R software. Next, the differentially expressed lncRNAs were identified as follows: FDR (adjusted p-value) <0.05 and |log2-fold change|>1. Differential expression analysis was performed using the "limma" package in R. In addition, a volcano plot was generated to show the upregulated and downregulated lncRNAs. The top10 downregulated and upregulated lncRNAs are shown in the heatmap.

To analyze the expression of SNHG16 and the prognosis of CRC patients, we performed overall survival analysis. First, TCGA-COAD RNA-seq was obtained from TCGA website, including 480 tumor tissues and 41 normal tissues (https://portal.gdc.cancer.gov/). Then, duplicated data or patients without survival information were excluded. Finally, a total of 467 samples enrolled in our survival analysis research.

Patient samples

The Research Ethics Committee approved this study of Wuhan University (Wuhan, Hubei, PR China). Informed consent was obtained from all participating patients. The enrolled patients were diagnosed with primary CRC by histopathologic diagnosis, underwent surgeries and had complete prognostic information. No patients received local or systemic neoadjuvant radiotherapy or/and chemotherapy and targeted therapy. One hundred and eleven human CRC tissues and PANTs (distance to cancer > 5 cm) were randomly obtained from patients in the Zhongnan Hospital of Wuhan University between January 2014 and December 2015. Each sample was snap-frozen in liquid nitrogen and then stored at -80°C.

Cell culture and treatment

The human colon cancer cell lines DLD1, Caco2, SW480, SW620, HT29, lovo, and HCT116 and the normal intestinal epithelium cell line NCM460 were obtained from the Cell Bank of Wuhan University. Cells were maintained in an incubator at 37°C and 5% CO_2. Cells were cultured in DMEM (Gibco, USA) supplemented with 2 mmol/L glutamine and 10% fetal calf serum (Gibco, USA).

supplementary material and method

Plasmid construction, siRNAs, miRNA, transfections, and infection.

miR-195-5p mimics and inhibitor (Table S2) were obtained from RiboBio Co. Ltd., China. The SNHG16 expression lentiviral vector (NR_038108.1), YAP1 expression lentiviral vector (NM_001130145), and TEAD1 lentiviral expression vector (NM_021961) were chemically synthesized, constructed, sequenced and identified by Shanghai GeneChem Chemical Technology, Co. Ltd. (Shanghai, China). The SNHG16 overexpression lentiviral vector (NR_038108.1) was chemically synthesized, constructed, sequenced and identified by Wuhan GeneCopoeia Technology, Co. Ltd. (Wuhan, China). The siRNA, shRNA, lentiviral vector of SNHG16, YAP1, and TEAD1 were chemically synthesized, constructed, sequenced and identified by Shanghai Genepharma Technology, Co. Ltd. The sequence of corresponding shRNA for plasmid construction was placed in supplementary table 1. For transfection, siRNA and plasmid were transfected into CRC cells using Lipofectamine 2000 transfection reagent (Invitrogen, Carlsbad, CA). For infection, we performed according to the manufacturer's instructions. First, we obtained the multiplicity of infection (MOI) values of HCT116 (MOI = 10) and DLD1 (MOI = 20) cells. For virus infection, virus and polybrene (final 7 μg/ml, Sigma Aldrich, Cat# 107689) were added to cells with 25% confluence. Fresh media was added 16 h after infection. The medium was changed to medium containing appropriate antibiotics 48 h after infection. After puromycin selection, the cells were maintained for at least one day without drug for further experiments.

qRT-PCR and subcellular fractionation

Total RNA was extracted from tissue samples and cell lines using a the TRIzol Reagent (Invitrogen, USA) according to the manufacturer's instructions. A Nanodrop2000 was used to quantify RNA concentration, and 1μg of total RNA was reverse transcribed into cDNA. We performed cDNA synthesis from mRNA by using the Primescript™ RT Reagent Kit (Vazyme, Nanjing, China; R223-01). cDNA synthesis for microRNA detection was carried out using miRNA 1st Strand cDNA Synthesis Kit (Vazyme, Nanjing, China; MR101-01/02). cDNA of microRNA was used for subsequent qRT-PCR using SYBR-Green PCR Master Mix (Vazyme, Nanjing, China; MQ101-01/02). cDNA of mRNA and lncRNA was used for subsequent qRT-PCR using SYBR-Green PCR Master Mix (Vazyme, Nanjing, China; Q311-02/03). The amplification conditions for cDNA of mRNA were as follows: 95°C for 2min, followed by 40 circles of 95°C for 5s, 60°C for 30s, and 72 °C for 30s. Each reaction was run on a BioRad IQ5 Real-time PCR instrument (BioRad, USA). Relative expression was calculated using the $2^{-\Delta\Delta Ct}$ method. The primer sequences are listed in Table S1 (Supplementary Table 1).

supplementary material and method

According to the manufacturer's instructions, cytosolic and nuclear fractions of HCT116 and DLD1 were isolated by the Nuclear/Cytoplasmic Extraction Kit (Cayman, USA). Subsequently, the RNA levels of a nuclear control transcript (U6), cytoplasmic control transcript (GAPDH), and SNHG16 were analyzed by qRT-PCR.

Protein extraction, western blotting, and protein quantification

Cells were lysed using RIPA buffer supplemented with complete proteinase inhibitor cocktail (Thermo Scientific, USA, Cat.No.89900). The detail of lyse process was introduced in the introduction of RIPA. Put it simply, we used 400ul of buffer per 25 cm^2 flask (or 150ul per well in six-well plate). Then, the lysate was homogenized using sonication, and the impurities were removed by high-speed centrifugation (13000rpm × 20min). Finally, the total proteins were quantified using the BCA reaction. Total proteins were separated by SDS-PAGE and subsequently transferred to PVDF membranes (Millipore, USA). Prior to incubation with diluted primary antibodies and HRP-conjugated secondary antibodies, blots were blocked in nonfat milk for an hour. The primary antibodies and dilution ratios were as follows: anti-E-cadherin (1:1000; CST, USA), anti-Vimentin (1:1000; Proteintech, China), anti-YAP1 (1:1000; Proteintech, China), and anti-GAPDH (1:5000; CST, USA). Quantitation of protein on all WB has been performed by Image J, and the results were normalized by the blank group. Finally, all the bar graphs of protein quantitation were placed on a supplementary file.

Luciferase reporter assay

For miR-195-5p target assay, the luciferase vector of YAP1 were chemically synthesized, constructed, sequenced and identified by RiboBio Technology, Co. Ltd. (Guangzhou, China) [1]. Likewise, SNHG16 fragments containing the putative binding sites for miR-195-5p were also synthesized, constructed, sequenced and identified by RiboBio Technology, Co. Ltd. (Guangzhou, China). Put it simply, the 3'-UTR sequences of YAP1 (WT and mut) and miR-195-5p binding sites in SNHG16 (WT and mut) were amplified from genomic DNA (human source). Then, the purified products of PCR products were connected to the pmiR-RB-Report plasmid (Promega, USA), which has similar cleavage sites of restriction enzyme with PCR products (5': Xhol; 3': Notl), with DNA ligase. Following transformation of Escherichia *coli* with recombinant plasmid DNA, the positive bacterial colony was screened by Ampicillin. The sequence and primers used in this process had been placed in supplementary table 1. For the SNHG16 promoter assay, the SNHG16 promoter (-2000/+1) and

modified constructs (-1861/+1, -1794/+1, -1368/+1) were amplified from the genomic DNA (human source) and inserted into the pGL3-Basic plasmid (Promega, USA). Then, the cDNA of the SNHG16 promoter containing the predicted TEAD1 binding sites was subcloned into the pGL3-Basic vector (Promega, USA), termed PGL3-SNHG16-pro (RiboBio Co. Ltd., China). Then, the TEAD1 binding site mutated SNHG16 promoter was synthesized by RiboBio (RiboBio Co. Ltd., China), termed as pGL3-SNHG16-pro-mut (1 and 2). According to the manufacturer's instructions, the transfection was carried out using Lipofectamine 2000, and luciferase activities were determined using the dual-Luciferase reporter system (Promega, USA).

Colony formation, CCK8, and wound healing assay

For colony formation detection, HCT116 cells were placed in 6-well plates at a density of 1000 cells per well. In the same way, DLD1 cells were placed at a density of 1000 cells per well. After two weeks of culture in an incubator, the cells were fixed with 4% paraformaldehyde and stained with 0.5% crystal violet. To perform a CCK8 assay, we seeded the cells in 96-well plates with the cell density of 3.0×10^3 cells/well in 100μL DMEM. After incubating for 0h, 24h, 48h, 72h, 96h, each well was put in 10μL CCK8 (Biosharp, China) and incubated for 2h at 37°C. Finally, the OD values of cells was measured at a wavelength of 450 nm by Multiskan FC microplate absorbance reader (Thermo Fisher, USA). A wound healing assay was also used to measure the migratory capacity of CRC cells. Cells were seeded in 6-well plates and allowed to grow until the confluence reached 90%. A 20 ul pipette tip was used to scrap several lines across the cell surface, and then plates were washed three times to remove nonadherent debris. Finally, the cells were incubated at 37°C with a serum-free medium, and photos were taken every 12 hours. The data analysis and process of wound healing are similar as Simona Martinotti [2]

Transwell migration and invasion assay

Twenty-four-well Transwell (8 μm pore size; Corning, USA) were used to assess the cell migration and invasion ability of CRC cells. For migration assay, 1.0 x 10^5 cells in 0.4 ml serum-free DMEM medium were seeded on the upper surface of the well. For the invasion assay, Transwell chambers were coated with Matrigel, and 1.5 x 10^5 cells were seeded on the upper surface of the chamber in 0.4 ml serum-free medium. After 36 - 48 h of incubation, cells on the upper surface of the chamber were removed with cotton swabs, and the remaining cells on the lower surface of the chamber were fixed and stained with 0.5% crystal violet solution.

Xenograft assays

supplementary material and method

The animal experiment was approved by the ethical committee of Zhongnan Hospital of Wuhan University and performed in accordance with the Guide for the Care and Use of Laboratory Animals of Wuhan University. To perform the tumor proliferation assay, 12 BALB/c nude mice (4-6 weeks old) (Hubei Research Center of Laboratory Animals, China) were randomly divided into three groups (n = 4 per group): HCT116/ Lv-anti-SNHG16 NC, HCT116/ Lv-anti-SNHG16 + Lv-Oe-YAP1 NC, and HCT116/ Lv-anti-SNHG16 + Lv-Oe-YAP1. In total, 5×10^6 HCT116 cells suspended in 200 ul medium were subcutaneously injected into the right flank of mice. After ten days, subcutaneous tumor size was measured by digital Vernier calipers every 1 week, and the subcutaneous tumor volume was calculated by the following formula: volume = $1/2 \times (\text{length} \times \text{width}^2)$. After thirty days, the transplantable tumor and 1ml mouse blood were collected for further research. For the metastasis experiment, the mice were randomly divided into three groups (n = 6 per group). The stable cell line was injected into the mice via the tail vein, and metastasis was assessed 30 days after injection by dissection. Finally, the mice were euthanized and necropsied to assess metastatic burden. Then, we further examined the tumor tissues, liver and lung tissues by hematoxylin and eosin (H&E) staining, Immunohistochemistry (IHC) staining and RT-PCR assay. Protein quantification in IHC was also represented by integral optic density (IOD), which is normalized by negative control group.

Statistical analysis

All the data were derived from at least three independent experiments, and p values < 0.05 were considered statistically significant. Through SPSS statistical software and GraphPad Prism software, all statistical analyses were performed. To assess the association between SNHG16 expression and YAP1, and miR-195-5p expression, we used Pearson's correlation analysis. Then, we applied the chi-square test to analyze the expression of SNHG16 and the clinicopathological status of CRC patients. Means of continuous outcome variables were appropriately tested with one-way analyses of variance or two-tailed Student's t-test. Groups of discrete variables were compared by means of the Mann-Whitney *U* test or Kruskal-Wallis nonparametric analysis of variance. In addition, Kaplan-Meier survival curves and log-rank tests were used for survival analysis. Finally, univariate and multivariate Cox-regression analyses were applied to identify the independent factors of prognosis.

supplementary tables

Supplementary Table 1: The sequences of the primers and shRNA

Gene	Primer Sequence (5' – 3')	
SNHG16	F: AGCAGAATGCCATGGTTTCC	R: GGTCAATTTAGGGCACGGTCT
TWIST1	F: CACTGAAAGGAAAGGCATCA	R: GGCCAGTTTGATCCCAGTAT
HMGA2	F: ACCCAGGGGAAGACCCAAA	R: CCTCTTGGCCGTTTTTCTCCA
FoxQ1	F: CACGCAGCAAGCCATATACG	R: CGTTGAGCGAAAGGTTGTGG
Snail2	F: CGAACTGGACACACATACAGTG	R: CTGAGGATCTCTGGTTGTGGT
Snail1	F: TCGGAAGCCTAACTACAGCGA	R: AGATGAGCATTGGCAGCGAG
PRX1	F: CCCGGATGCTTTTGTTCGAGA	R: CATGTGGCAGAATAAGTAGCCAT
ZEB2	F: AGGAGCAGGTAATCG	R: TGGGCACTCGTAAGG
ZEB1	F: CGAGTCAGATGCAGAAAATGAGCAA	R: ACCCAGACTGCGTCACATGTCTT
YAP1	F: CTTCTCCCGGGATGTCTCAG	R: TCAAGGTAGTCTGGGAAACGG
miR-195-5p RT	CTCAACTGGTGTCGTGGAGTCGGCAATTCAGTTGAGGCCAATAT	
miR-195-5p	F: CGCAGCACAGAAATATTGGC	R: CTCAACTGGTGTCGTGGAGTC
U6 RT	R: AACGCTTCACGAATTTGCGT	
U6	F: CTCGCTTCGGCAGCACA	R: AACGCTTCACGAATTTGCGT
TEAD1	F: TGCGCAAAGAAGAATGGTAGA	R: TGCGCAAAGAAGAATGGTAGA
ChIP primer site1	F: GAAAGGAACTATGTTTGCAGGGA	R: CATTCAGCAGTAATTCTGGCCA
ChIP primer site2	F: AGGAGAATCACTTGAGCTTGGG	R: GAAAGGAACTATGTTTGCAGGGA
TEAD1_s hRNA	5'-CACCGCTTGAATCAGTGGACATTCGCGAACGAATGTCCACTGATTCAAGC -3'	
SNHG16_s hRNA	5'-AATTCAAAAAAGCCTCTGCTGCTAATTGTTCTCTTGAAACAATTAGCAGCAGAGGCG -3'	
YAP1_s hRNA	5'-'CACCGCTTGAATCAGTGGACATTCGCGAACGAATGTCCACTGATTCAAGC -3''	
h-YAP1-3'UTR-WT	F: GCGCTCGAGTGCAGTTTTCAGGCTAATACAGA	R: AATGCGGCCGCAGGATACATTCCACTACCACACTA
h-YAP1-3'UTR-MUT	F: CCTTGTCCATACGACGAGTTAATGTATTGCTGACCTCTT	R: ATACATTAACTCGTCGTATGGACAAGGAAGAGCAAAATA
h-SNHG16 -3UTR WT	F: GCGCTCGAGTAGCTCCCAGCGATGCCAGA	R: AATGCGGCCGCCACTGATATTCTATGCAAAGATCTG
h-SNHG16 -3UTR MUT	F: CCTGCCTCACGACGAAATTGTTCCTCTAAAGTAATCG	R: GAACAATTTCGTCGTGAGGCAGGGCTGTGCTGA

supplementary tables

Supplementary Table 2: The sequence of miR-195-5p inhibitor and mimics

Gene	Primer Sequence (5' – 3')
Mimics	Sense : UAGCAGCACAGAAAUAUUGGC<br>Antisense : GCCAAUAUUUCUGUGCUGCUA
Mimics NC	Sense: UUUGUACUACACAAAAGUACUG<br>Antisense: CAGUACUUUUGUGUAGUACAAA
Inhibitor	GCCAAUAUUUCUGUGCUGCUA
Inhibitor NC	CAGUACUUUUGUGUAGUACAAA

supplementary figures

Supplementary Fig 1. The ectopic expression of SNHG16 affects proliferation and EMT of CRC cells

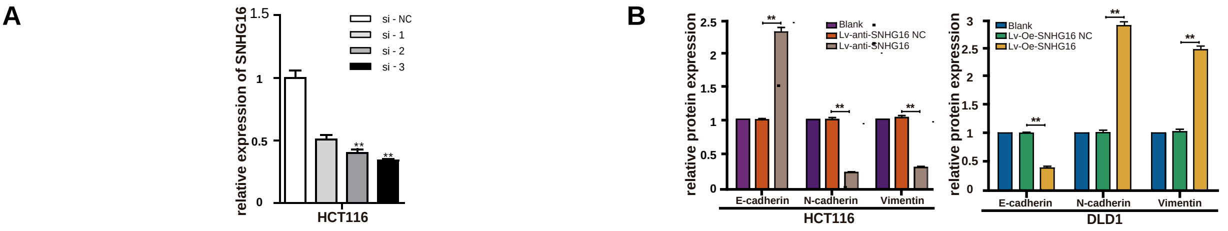

A: The knockdown effect of SNHG16 siRNA was validated by qRT-PCR. **B:** The protein quantitation of EMT markers expression from SNHG16 overexpression or knockdown CRC cell lines were shown as bar graphs. Statistical analysis was conducted using one-way ANOVA. $^{**}p < 0.01$; Error bars, SEM.

Supplementary Fig 2. SNHG16 facilitates CRC cellular proliferation, migration, and EMT in a YAP1-dependent manner.

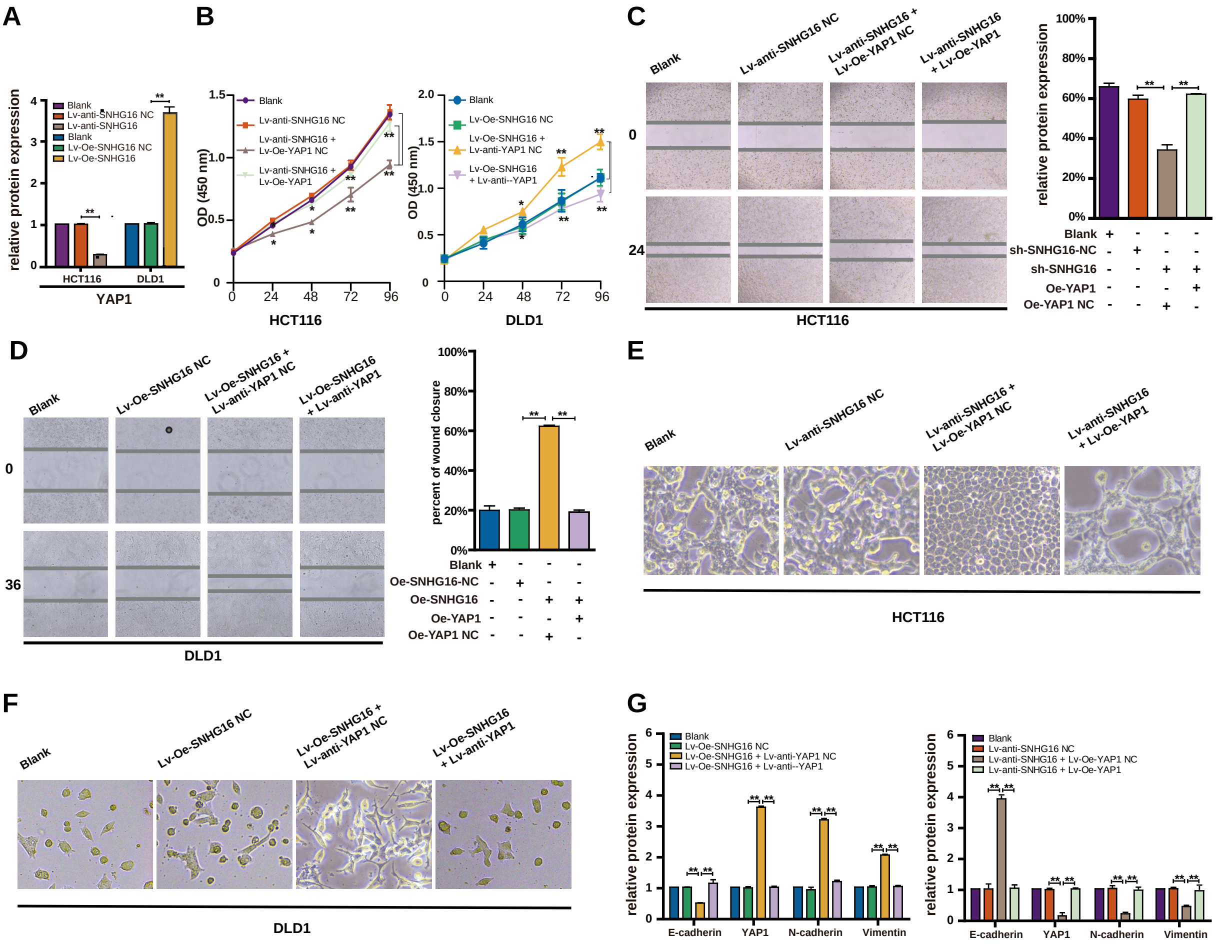

A: The protein quantitation of YAP1 expression from SNHG16 overexpression or knockdown CRC cell lines were shown as bar graphs. **B:** CCK8 cell proliferation assay in HCT116 (HCT116^{Blank}, HCT116$^{\text{Lv-anti-SNHG16-NC}}$, HCT116$^{\text{Lv-anti-SNHG16 + Lv-Oe-YAP1-NC}}$, and HCT116$^{\text{Lv-anti-SNHG16 + Lv-Oe-YAP1}}$) and DLD1 (DLD1^{Blank}, DLD1$^{\text{Lv-Oe-SNHG16 NC}}$, DLD1$^{\text{Lv-Oe-SNHG16 + Lv-anti-YAP1-NC}}$, DLD1$^{\text{Lv-Oe-SNHG16 + Lv-anti-YAP1}}$). **C:** The migration in HCT116^{Blank}, HCT116$^{\text{Lv-anti-SNHG16-NC}}$, HCT116$^{\text{Lv-anti-SNHG16 + Lv-Oe-YAP1-NC}}$, and HCT116$^{\text{Lv-anti-SNHG16 + Lv-Oe-YAP1}}$ was detected by wound healing assay. **D:** The migration in DLD1^{Blank}, DLD1$^{\text{Lv-Oe-SNHG16 NC}}$, DLD1$^{\text{Lv-Oe-SNHG16 + Lv-anti-YAP1-NC}}$, and DLD1$^{\text{Lv-Oe-SNHG16 + Lv-anti-YAP1}}$ was detected by wound healing assay. All representative data are from three independent experiments. **E:** Photomicrographs of HCT116^{Blank}, HCT116$^{\text{Lv-anti-SNHG16-NC}}$, HCT116$^{\text{Lv-anti-SNHG16 + Lv-Oe-YAP1-NC}}$, and HCT116$^{\text{Lv-anti-SNHG16 + Lv-Oe-YAP1}}$. **F:** Photomicrographs of DLD1^{Blank}, DLD1$^{\text{Lv-Oe-SNHG16 NC}}$, DLD1$^{\text{Lv-Oe-SNHG16 + Lv-anti-YAP1-NC}}$, DLD1$^{\text{Lv-Oe-SNHG16 + Lv-anti-YAP1}}$. **G:** The protein quantitation of YAP1 and EMT markers expression from eight different cell lines were shown as bar graphs. Statistical analysis was conducted using one-way ANOVA. Error bars, SEM. $^{**}p < 0.01$.

supplementary figures

Supplementary Fig 3. miR-195-5p could potently abrogate the effect of SNHG16/YAP1 axis on tumor progression.

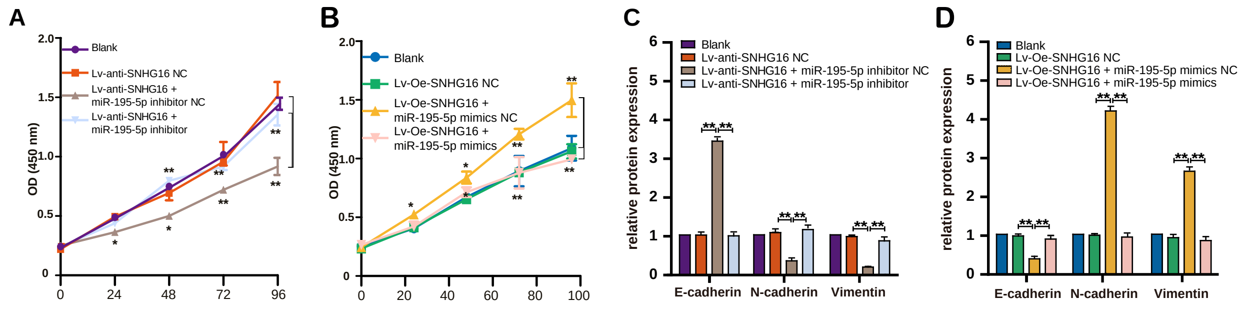

A-B: CCK8 cell proliferation assay in HCT116 (A) and DLD1 (B). **C:** The protein quantitation of EMT markers expression from HCT116 ^{Blank}, HCT116 $^{Lv\text{-}anti\text{-}SNHG16\text{-}NC}$, HCT116 $^{Lv\text{-}anti\text{-}SNHG16 + miR\text{-}195\text{-}5p\ inhibitor\ NC}$ and HCT116 $^{Lv\text{-}anti\text{-}SNHG16 + miR\text{-}195\text{-}5p\ inhibitor}$ were shown as bar graphs. **D:** The protein quantitation of EMT markers expression from DLD1 ^{Blank}, DLD1 $^{Lv\text{-}Oe\text{-}SNHG16\text{-}NC}$, DLD1 $^{Lv\text{-}Oe\text{-}SNHG16 + miR\text{-}195\text{-}5p\ mimics\ NC}$, and DLD1 $^{Lv\text{-}Oe\text{-}SNHG16 + miR\text{-}195\text{-}5p\ mimics}$ were shown as bar graphs. Statistical analysis was conducted using one-way ANOVA. Error bars, SEM. **p < 0.01

Supplementary Fig 4. SNHG16 functions as a ceRNA and sponges miR-195-5p, further regulating YAP1 expression .

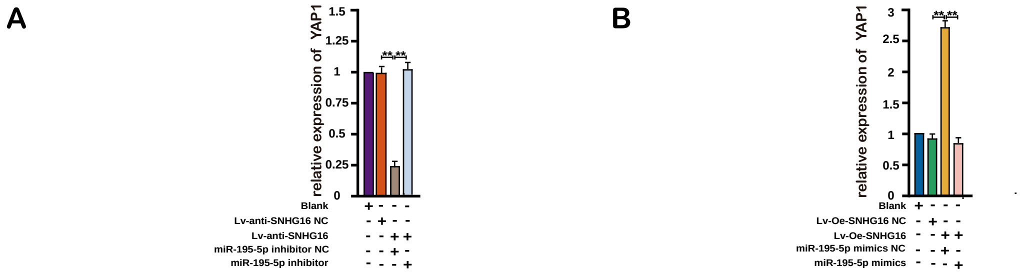

A: The protein quantitation of YAP1 expression from HCT116 ^{Blank}, HCT116 $^{Lv\text{-}anti\text{-}SNHG16\text{-}NC}$, HCT116 $^{Lv\text{-}anti\text{-}SNHG16 + miR\text{-}195\text{-}5p\ inhibitor\ NC}$ and HCT116 $^{Lv\text{-}anti\text{-}SNHG16 + miR\text{-}195\text{-}5p\ inhibitor}$ were shown as bar graphs. **B:** The protein quantitation of YAP1 expression from DLD1 ^{Blank}, DLD1 $^{Lv\text{-}Oe\text{-}SNHG16\text{-}NC}$, DLD1 $^{Lv\text{-}Oe\text{-}SNHG16 + miR\text{-}195\text{-}5p\ mimics\ NC}$, and DLD1 $^{Lv\text{-}Oe\text{-}SNHG16 + miR\text{-}195\text{-}5p\ mimics}$ were shown as bar graphs. Statistical analysis was conducted using one-way ANOVA. Error bars, SEM. **p < 0.01.

supplementary figures

Supplementary Fig 5. The effect of YAP1 on tumor colony formation, migration, invasion, and EMT of CRC

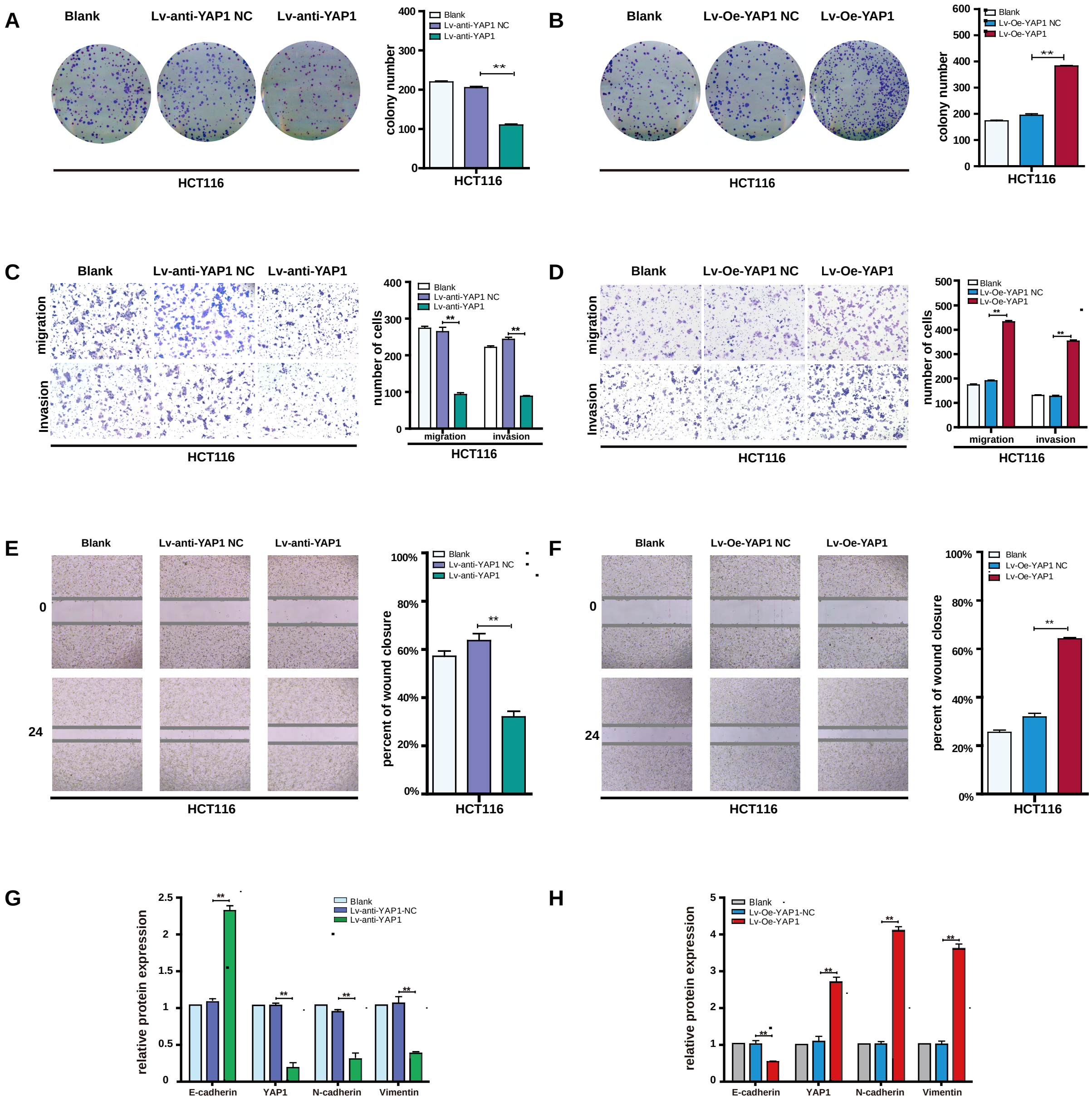

A-B: Colony formation assay was performed in CRC cell lines following YAP1 knockdown or overexpression. **C-D:** Graphical representation of transwell showing migration and invasion ability of HCT116 after YAP1 knockdown (C) or overexpression (D). **E-F:** Wound healing assay was performed in CRC cell lines following YAP1 knockdown (E) or overexpression (F). **G-H:** The quantitation of EMT markers expression from YAP1 knockdown (G) or overexpression (H) CRC cell lines was shown as bar graphs. All representative data are from three independent experiments. Statistical analysis was conducted using one-way ANOVA. Error bars, SEM. $**p < 0.01$.

supplementary figures

Supplementary Fig 6. The effect of YAP1-SNHG16 positive feedback loop on the progression of tumor.

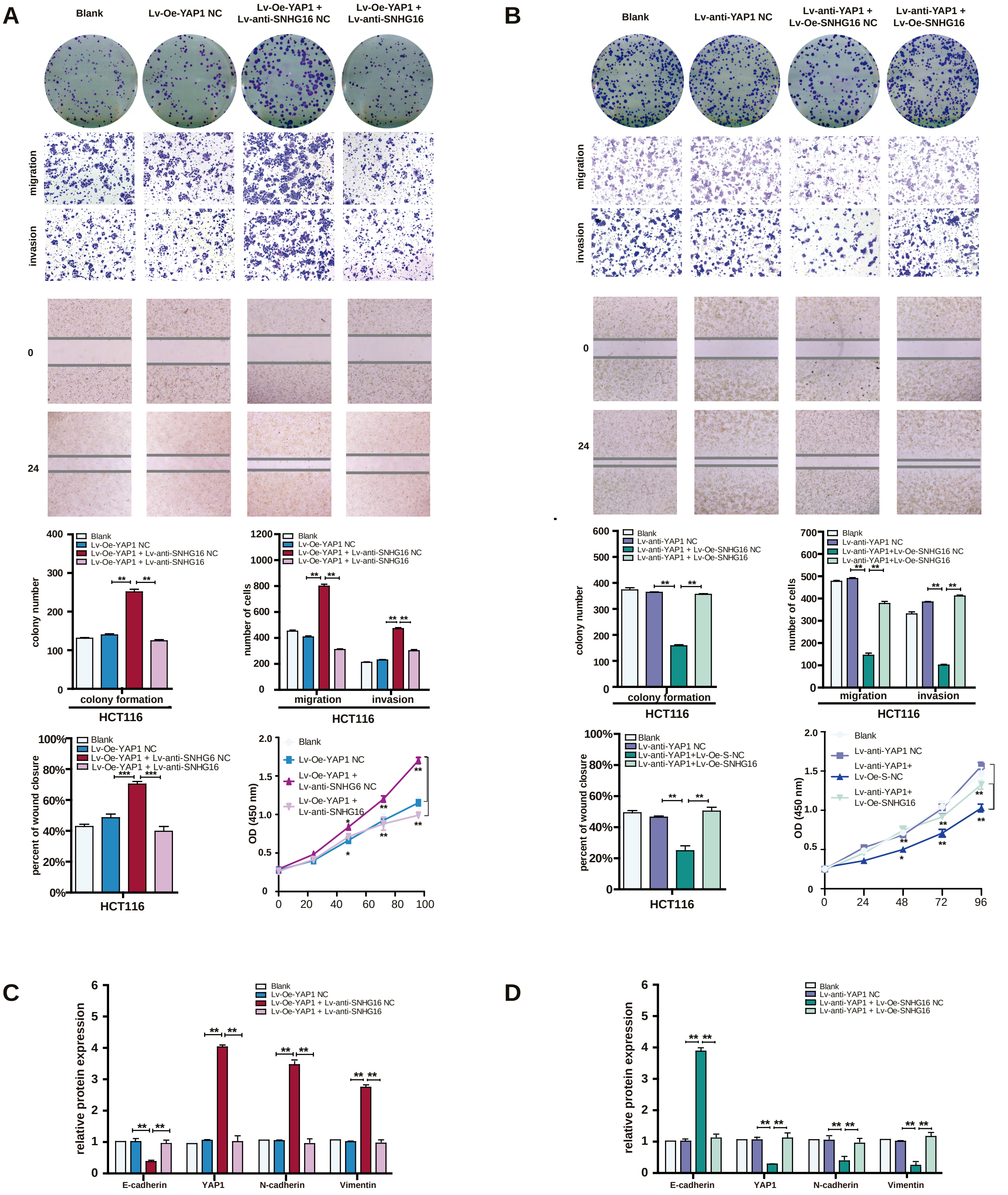

A: The colony formation, migration, invasion and proliferation in HCT116^{Blank}, HCT116$^{\text{Lv-Oe-YAP1-NC}}$, HCT116$^{\text{Lv-Oe-YAP1 + Lv-anti-SNHG16-NC}}$, and HCT116$^{\text{Lv-Oe-YAP1 + Lv-anti-SNHG16}}$ were detected by colony formation, wound healing, transwell assay, and cck8. **B:** Colony formation, cck8, wound healing, and transwell assay were performed in HCT116^{Blank}, HCT116$^{\text{Lv-anti-YAP1-NC}}$, HCT116$^{\text{Lv-anti-YAP1 + Lv-Oe-SNHG16-NC}}$, and HCT116$^{\text{Lv-anti-YAP1 + Lv-Oe-SNHG16}}$. **C:** The quantitation of protein expression from HCT116^{Blank}, HCT116$^{\text{Lv-Oe-YAP1-NC}}$, HCT116$^{\text{Lv-Oe-YAP1 + Lv-anti-SNHG16-NC}}$, and HCT116$^{\text{Lv-Oe-YAP1 + Lv-anti-SNHG16}}$ were shown as bar graphs. **D:** The quantitation of protein expression from HCT116^{Blank}, HCT116$^{\text{Lv-anti-YAP1-NC}}$, HCT116$^{\text{Lv-anti-YAP1 + Lv-Oe-SNHG16-NC}}$, and HCT116$^{\text{Lv-anti-YAP1 + Lv-Oe-SNHG16}}$ were shown as bar graphs. All representative data are from three independent experiments. Statistical analysis was conducted using one-way ANOVA. Error bars, SEM.**p < 0.01.

supplementary figures

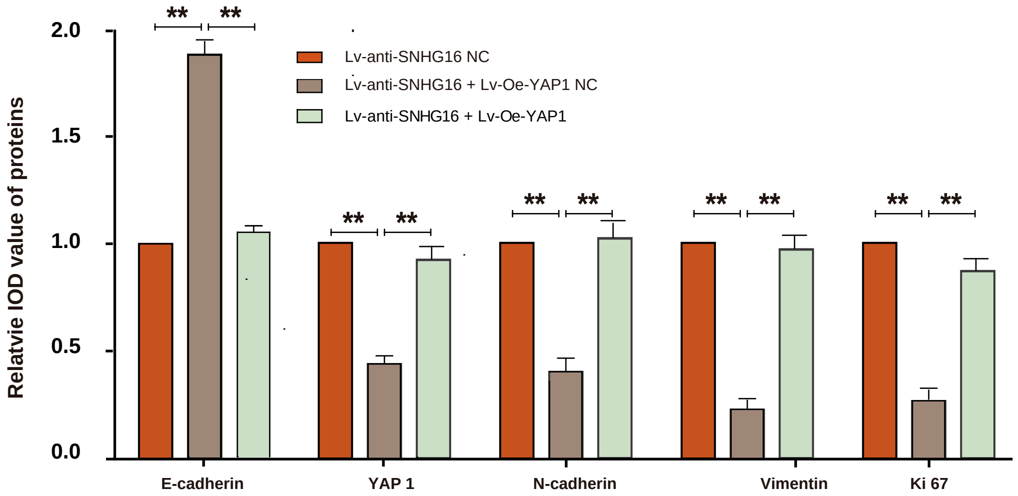

Supplementary Fig 7: Comparison of relative integrated optical density (IOD) values of Ki67 and EMT related markers among HCT116 $^{\text{Lv-anti-SNHG16-NC}}$, HCT116 $^{\text{Lv-anti-SNHG16 + Lv-Oe-YAP1 NC}}$, HCT116 $^{\text{V-anti-SNHG16 + Lv-Oe-YAP1}}$. All representative data are from three independent experiments. Statistical analysis was conducted using one-way ANOVA. Error bars, SEM. **p < 0.01.

REFERENCE

1 Sun M, Song HB, Wang SY, et al. Integrated analysis identifies microRNA-195 as a suppressor of Hippo-YAP pathway in colorectal cancer. *Journal of hematology & oncology*. 2017; 10.

2 Martinotti S, Ranzato E. Scratch Wound Healing Assay. *Methods in molecular biology (Clifton, NJ)*. 2020; 2109: 225-229.
